# The bacterial community of Quesnel Lake sediments impacted by a catastrophic mine tailings spill differ in composition from those at undisturbed locations – two years post-spill

**DOI:** 10.1038/s41598-019-38909-9

**Published:** 2019-02-25

**Authors:** I. Hatam, E. L. Petticrew, T. D. French, P. N. Owens, B. Laval, S. A. Baldwin

**Affiliations:** 1 Department of Chemical and Biological Engineering, University of British Columbia, Vancouver, British Columbia V6T1Z3 Canada; 2 Geography Program and Quesnel River Research Centre, University of Northern British Columbia, Prince George, British Columbia V2N4Z9 Canada; 3 Environmental Science Program and Quesnel River Research Centre, University of Northern British Columbia, Prince George, British Columbia V2N4Z9 Canada; 4 Department of Civil Engineering, University of British Columbia, Vancouver, British Columbia V6T1Z3 Canada

## Abstract

The West Basin of Quesnel Lake (British Columbia, Canada) suffered a catastrophic disturbance event in August 2014 when mine tailings and scoured natural material were deposited into the lake’s West Basin due to an impoundment failure at the adjacent Mount Polley copper-gold mine. The deposit covered a significant portion of the West Basin floor with a thick layer of material. Since lake sediments host bacterial communities that play key roles in the geochemical cycling in lacustrine environments, it is important to understand which groups inhabit the newly deposited material and what this implies for the ecological function of the West Basin. Here we report a study conducted two years post-spill, comparing the bacterial communities from sediments of both disturbed and undisturbed sites. Our results show that sediments from disturbed sites differed in physical and chemical properties than those in undisturbed sites (e.g. higher pH, particle size and Cu concentration). Furthermore, bacterial communities from the disturbed sites appeared to be legacy communities from the tailings impoundment, with metabolic potential revolving mainly around the cycling of S and metals, whereas the ones from the undisturbed sites were associated with the cycling of N.

## Introduction

Mining and mineral processing produce large quantities of waste material. Depending on the type of metal to be extracted, between 60% and 99.99% of the mined ore ends up as waste^1^. A major form of this waste is tailings, a mix of finely crushed and ground rock and processing fluids that remain after extraction of the valued resource^2^. Tailings are most often stored in large dammed impoundments, and it is estimated that there are approximately 3500 of these worldwide^2^. Although most tailings storage facilities are effective, there has been an alarming failure rate of between 1:700 – 1:1750^2^. When tailing spills occur, impacts on receiving environments can be catastrophic, especially if these are pristine aquatic ecosystems. Immediate impacts are very apparent and can include human casualties and damage to flora and fauna due to the sheer volume of the spill. But potential longer-term risks of the deposited tailings to the ecosystem are more difficult to ascertain. When tailings are deposited into lakes, disruption of the aquatic food chain is of concern since this can impact economically important species such as fish^2^. Lower trophic level organisms such as sediment microorganisms are often ignored when assessing environmental impacts even though they play a key role in nutrient and geochemical cycling that can impact the whole lake ecosystem. Thus, studying the impacts of mine tailings spills on lake sediment bacterial communities is of high importance.

**Figure 1:**
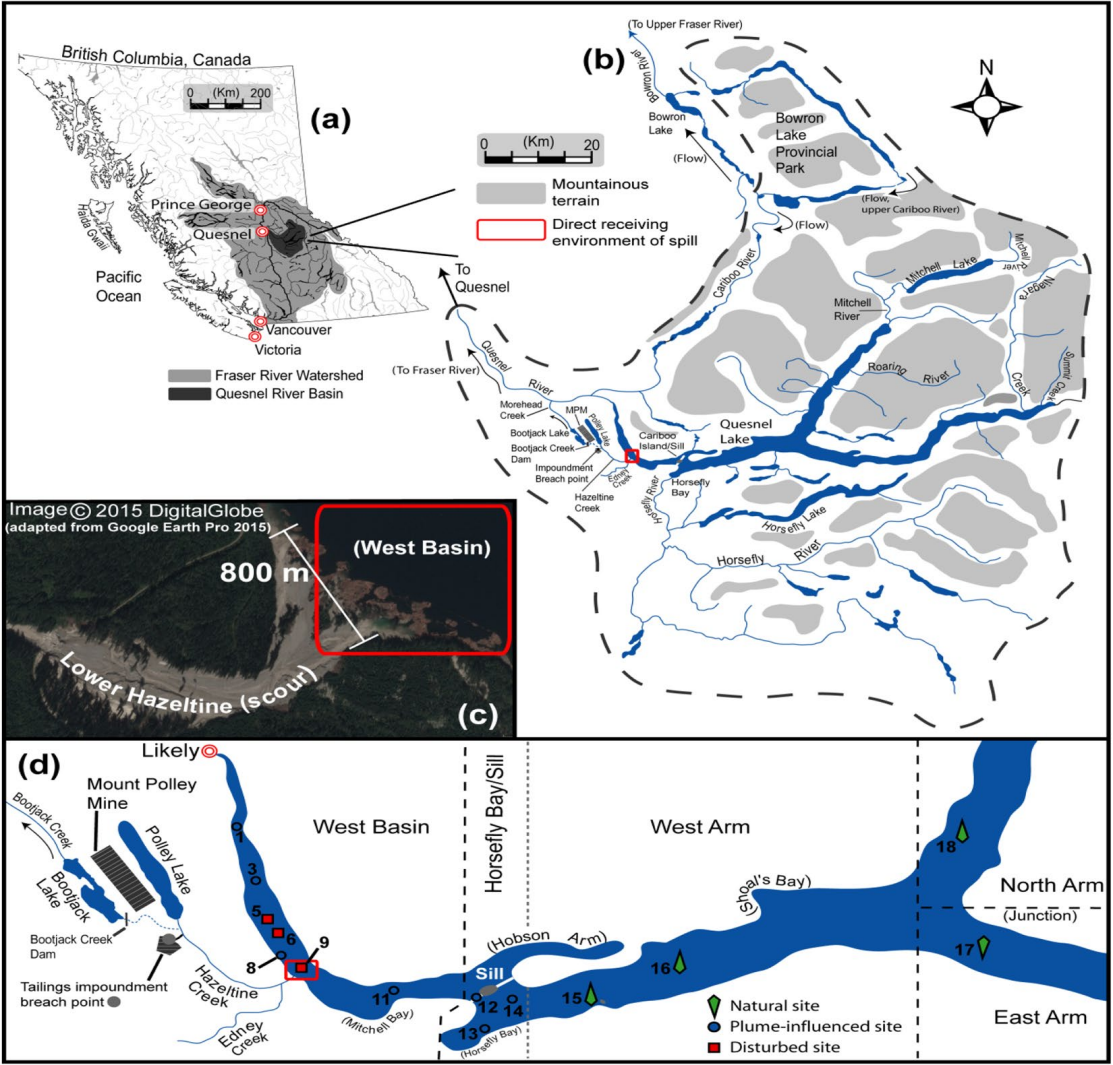
The study area and sites sampled with a slo-corer in July 2016. (**a**) The position of the Quesnel River basin in relation to mainland British Columbia, Canada, and the Fraser River watershed. (**b**) Major sub-basins that drain into Quesnel Lake, and the outlet of Quesnel Lake (Quesnel River) located at the village of Likely (see (d)). (**c**) A 2015 aerial view of the entry point of spill-related materials to Quesnel Lake (West Basin, which is the west portion of the West Arm of the lake). (**d**) Slo-core sampling sites in Quesnel Lake shown in relation to the direct receiving environment (West Basin at Hazeltine Creek) of spill-related materials. Satellite imagery was created using Google earth pro. Line map was adopted from Energy, Mines and Resources Canada, Surveys and Mapping Branch, Ottawa, ON, Canada (1973, 1974, 1975, 1976, and 1982). 1:50,000 scale Polyconic Projections.

In this present study we investigated the medium-term effect that the catastrophic tailings spill from the Mount Polley Mining Corporation (MPMC) mine site had on the bacterial communities on the sediments in Quesnel Lake. The MPMC mine site is located 9.2 km upstream and 200 m above Quesnel Lake, a pristine fjord-type lake with three arms (West, East and North, Fig. 1). On August 4^th^ 2014, a portion of the retaining wall of the tailing storage facility impoundment on the MPMC mine site failed. This led to a release of 10.6 M m^3^ of supernatant water, 7.3 M m^3^ of tailings solids, 6.5 M m^3^ of interstitial water, and 0.6 M m^3^ of construction materials^3^. Most of this material flowed down Hazeltine Creek channel, scouring additional material along the way, and this mixture was deposited into Quesnel Lake’s West Basin, the west portion of the West Arm, which extends 20 km from a 35 m deep sill at Cariboo Island, to the Quesnel River outflow (annotated in Fig. 1)^4^. The surge of material generated an extensive lake bottom deposit, consisting of tailings and eroded overburden, measuring 5.5 km wide, from 1 m to more than 10 m deep, and up to 1.2 km across the West Basin^3,4^. Furthermore, a plume of turbidity composed of small (D_50_ ~ 1 μm) suspended particles was observed expanding both westward towards the mouth of the Quesnel River and eastwards reaching parts of the West Arm beyond the sill^4^. This indicates that spill-associated material might cover a larger area of the lake’s floor than the original deposition area, essentially covering parts of the West Arm with a newly created lake floor, replacing the natural lake sediments.

Natural lake sediments are a heterogeneous mix of organic matter, biota, and mineral phases that come both from autochthonous and allochthonous sources, that are constantly deposited and buried^5–8^. Natural lake sediments host highly abundant and active microbial communities spanning all three domains of life. However, the bulk of the activity takes place in the upper 5 cm of the sediment, and at the sediment–water interface^9,10^. Bacteria have been reported to be the numerically dominant component of the microbial community of lake sediments at depths of up to 10 cm and account for the bulk of the metabolic activity in the sediment^9–13^. Bacterial transformation of both organic and non-organic material in lake sediments impacts the cycling of many elements (such as C, N, S, P and metals) in both the sediment and the water column, due to sediment resuspension at the sediment–water interface, and upwelling^13–16^. Thus, it is clear that the bacterial communities found in lake sediments provide important ecological services to lacustrine ecosystems. In contrast to natural lake sediments, tailings produced in the process of metal mining are mainly composed of finely crushed rock, and are characterized by low organic matter content and the presence of residual metals^17,18^. Furthermore, the biogeochemical cycles in metal mine tailings mainly involve the reduction of S and the redox transformation of metals^19,20^.

Given the key role that bacterial communities play in lacustrine sediment biogeochemistry, it is important to understand the impact that the deposition of tailings and associated scoured material had on the sediment bacterial community in Quesnel Lake’s West Basin. We collected sediment cores from 14 different sites across all three arms of Quesnel Lake two years post-spill (Fig. 1). Three sites were in close proximity to the Hazeltine Creek entrance to Quesnel Lake (within the initial deposition zone) and were defined as disturbed sites. Seven coring sites were within the spatial extent of the 2014 plume and were initially classified as plume-influenced sites (i.e. mild to light disturbance) and four sites were in areas not reached by the plume and were classified as natural (or undisturbed) sites. We sequenced the V4 region of the 16 S rRNA gene to compare the composition of the bacterial communities from these three groups of sites and correlated the community composition to the chemical and physical attributes of the three groups. This was done in order to determine the effect that these attributes had on the composition of the bacterial community.

## Results

### Chemical and physical profiles of the cores

**Figure 2:**
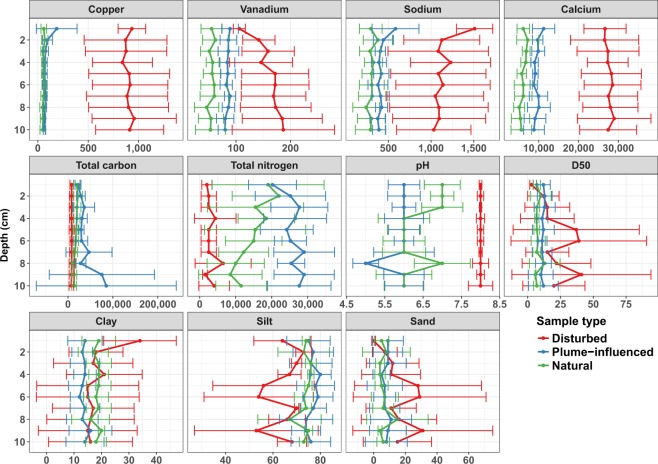
Concentration of important metals and metalloids, carbon and nitrogen, and the particle size composition of the sediment core samples. Sites were grouped to either Disturbed, Plume-influenced, or Natural (Undisturbed). Error bars represent standard deviation from the mean. Metal, metalloid, C, and N concentrations are in mg/Kg dry weight, D_50_ is the median particle size in µm, clay represents percent of particles between 0 µm–2 µm in diameter, silt represents percent of particles between 2.01 µm and 62.5 µm in diameter, total sand represents particles >62.5 µm.

Cores from the disturbed sites differed in particle size distribution from plume-influenced and natural sites (Figs 2, S1). Generally, the median particle diameter (D_50_) increased with depth in cores from disturbed sites, while showing no depth related trends in cores from plume-influenced and natural sites (Fig. 2). The average D_50_ ranged from 3.2 μm–40 μm in sections for the disturbed cores, whereas the range in plume-influenced and natural sites was 10.6 μm–12.6 μm and 6.99 μm–11.6 μm, respectively (Fig. 2). Similarly, the percentage of particles classified as sand-sized (>62.5 μm) was highest in sections deeper than 3 cm in the disturbed cores (Figs 2, S1). For both particle size distribution and median particle size value, there was greater variability within sections of different cores from disturbed sites than in those from other site types (Fig. 2).

We examined the bulk concentrations of various metals and metalloids, as well as total C and total N, and the pH of the pore-water, in each of the core sections (Figs 2, S1). While there were minimal visual or statistical depth-related trends in the measurements of these parameters in cores from any of the site types, core sections from disturbed sites differed in many of them from those of the plume-influenced and natural sites (Figs 2, S1). Most significant of those, was the difference in Cu concentrations, which were up to two orders of magnitude higher in samples from disturbed sites than the others. However, there were also large differences in the concentrations of V, Ca, and Na (Fig. 2) as well as other elements (e.g. B, Mo, Sr, Ti, and Zr; Fig. S1). The pore-water pH levels were also higher in core sections from disturbed sites averaging around 8.3, whereas at both the plume-influenced and natural sites, pH levels were circumneutral (6.5–7.5, Figs 2, S1). Total C and total N concentrations were higher in the core sections from plume-influenced and natural sites compared to those from the disturbed sites, both being as high as five orders of magnitude greater in the plume-influenced sites versus the disturbed sites (Figs 2, S1).

**Figure 3:**
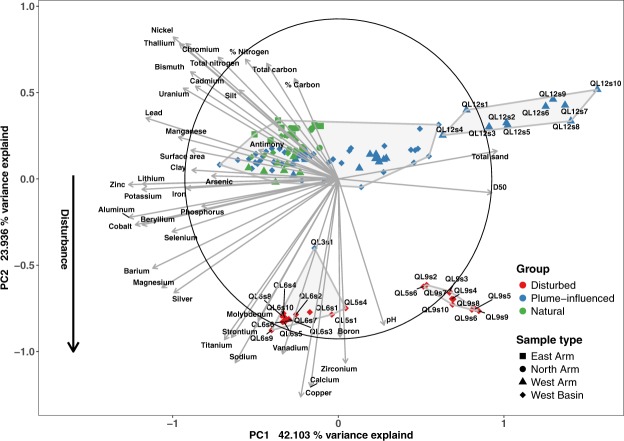
PCA ordination scatter plot of the samples based on Manhattan distances on metal and metalloid concentrations. Polygons represent grouping of samples based on hierarchical clustering, number of clusters determined using *K*-means clustering. Clustering significance was tested using MRPP (A = 0.487, p < 0.001). The circle represents the equilibrium contribution of chemical and physical parameters to the separation of the samples in two-dimensional space (i.e. parameters with arrows larger than the radius of the circle, contribute more than the average of all parameters to the separation of the samples). QL followed by number denotes site number as shown in Fig. 1, s followed by a number denotes section number as cm from the top.

When looking at the physico-chemical profiles within all cores taken together, using multivariate statistics (Fig. 3), there is a clear separation along axis 2 of the PCA between samples from disturbed sites and samples from plume-influenced and natural sites (Fig. 3). According to *K*-means clustering, four clusters were optimal to explain the numbers of groups the samples group into (Fig. S2), and the association of samples to the four groups was done using UPGMA (Fig. S3). Those groups were then overlaid on the PCA plot. Based on this, all core sections from the disturbed sites clustered into two statistically significant groups (MRPP, *A* = 0.487, *p* value < 0.001). All sections from site 9 along with one section from site 5 were grouped together while the rest of the sections from site 5, all the core sections from site 6 along with the top 1 cm section from site 3 which is a plume-influenced site found in close proximity to the disturbed sites, were clustered into the other disturbed group (Fig. 3). The PCA indicated that the factor separating the two disturbed groups was particle size, with site 9 having a larger median particle size (Fig. 3). Not surprisingly, Cu was amongst the metals and metalloids that contributed to the separation between the disturbed sites and the rest of the groups (Fig. 3). However, Ca and Na as well as total N concentrations also contributed highly to the separation of the disturbed sites from the other groups (Fig. 3). The undisturbed and plume-influenced sites clustered into two distinct groups. Plume-influenced sites from both sides of the sill clustered into one group, without clear separation between samples (Fig. 3). Sections from site 12, which is on the shallow sill separating the West Basin from the rest of the West Arm, clustered as a separate group due to differences in particle size (Fig. 3). Measurements of pH, PO_4_^3−^, N-NH_3_, N-NO^−^_3_, N-NO^−^_2_ and dissolved Fe in water at the sediment–water interface showed no significant differences in any of the parameters measured between any of the sites, with most sites having PO_4_^3−^, N-NO_2_^−^, N-NO_3_^−^ and Fe below the analytical detection limits (Table S1). The pH of the water at the sediment–water interface was 7 (Table S1).

### Composition of the bacterial communities

The bacterial communities in all core sections were classified into over 80 class-level groups, of varying degrees of relative abundance. In order to examine the more dominant taxa, we focused on classes with relative abundance of >5% of the total number of sequences per sample (Fig. S4). For the most part, similar class level taxonomic groups dominated all samples from all site types (i.e. natural, plume-influenced and disturbed). However, while operational taxonomic units (OTUs) classified as members of the classes Deltaproteobacteria (5.3–15.6%), Betaproteobacteria (11–21.5%), and Flavobacteria (5.2–28.1%) were much more abundant in the disturbed samples, OTUs classified as members of the different classes of the phylum level groups Actinobacteria (5.1–15.9%) and Acidobacteria (5.7–16.5%) were dominant in the natural and plume-influenced sites (Fig. S4). However, it should be noted that most of the OTUs found in the samples did not classify below class level and indeed a large proportion of the OTUs could not be classified past the domain level (up to 40% of the sequences, the “Others” group in Fig. S4). This is especially the case for samples from the plume-influenced and natural sites which had a maximum 70% of the sequences classify only at the domain level and an average of 40%, as opposed to samples from disturbed sites which only had up to 25% of the sequences classify only at the domain level and an average of 17.6% (Fig. S4).

**Figure 4:**
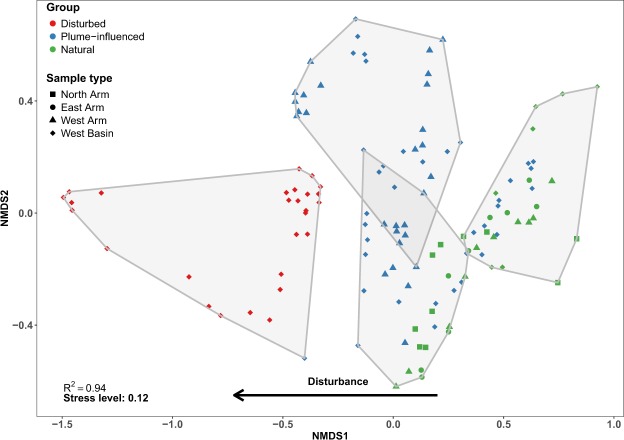
NMDS ordination plot of the samples based on weighted Bray-Curtis distances on OTU data. Polygons represent grouping of samples based on hierarchical clustering, number of clusters determined using *K*-means clustering. Clustering significance was tested using AMOVA (F_3,125_ = 21.06, *p* value < 0.001).

**Figure 5:**
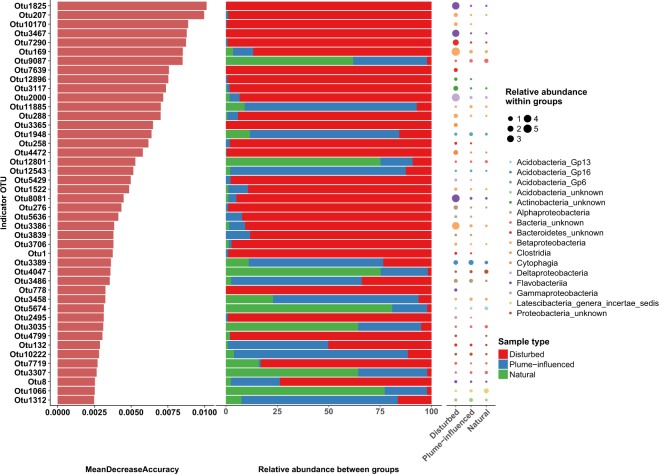
Indicator OTUs for the separation of site based on the classification as disturbed, plume-influenced, natural using a random forest model. Cutoff for OTU importance was chosen based on a broken stick model on mean decrease in accuracy. Relative abundance between groups denoted the percent of sequences for each OTU that came from each group. Relative abundance within group denotes how abundant each OTU (percent of total sequences) was in a group.

We examined the bacterial community in the sediments by looking at the composition as a whole in a taxonomic-independent manner, using non-metric multidimensional scaling (NMDS) with weighted Bray-Curtis as a dissimilarity measure. Based on the community composition, the samples clustered into four statistically significant groups (Fig. 4, AMOVA F_3,125_ = 21.06, *p* value < 0.001). Similarly, to the clustering based on metals and metalloids concentrations, samples from the disturbed sites along with the top 1 cm section from site 3 clustered into one group and there was a clear trajectory of separation between disturbed and undisturbed sites along NMDS axis1 (Fig. 4). While samples collected at the sill (identified as West Basin) clustered into a single distinct group based on the chemistry data, this was not the case based on bacterial community composition (Fig. 4). For the most part, sections for the same core clustered into the same group (Fig. S5), and there were no depth-related trends between groups (e.g. clusters that are composed of only top sections, or only bottom sections). According to a random-forest based analysis to identify indicator OTUs, there were 44 OTUs that best explained the separation of the disturbed samples from the plume-influenced and natural sites (Fig. 5). Many of these disturbed site indicator OTUs belonged to the classes Deltaproteobacteria, Betaproteobacteria, Flavobacteria as well as other classes within the phylum Bacteroidetes. Indicator OTUs assigned to these three taxonomic groups accounted for over 35% of the total number of sequences coming from disturbed site samples. Network analysis of OTUs from disturbed sites revealed 80 OTUs with statistically significant (*p* value < 0.05) and positive (*rho* > 0.5) co-occurrence patterns. Of these, 26 were indicator OTUs (out of the total 29 indicator OTUs, Fig. S6a). Furthermore, eight out of the 10 most central (defined as those with the greatest number of connections) nodes in the network were indicator OTUs. Lastly, 23 out of the 26 indicator OTUs grouped into their own sub-network (Fig. S6b), indicating potentially strong metabolic ties between indicator OTUs of the disturbed sites.

### Constraints of physico-chemical profile on microbial community

**Figure 6:**
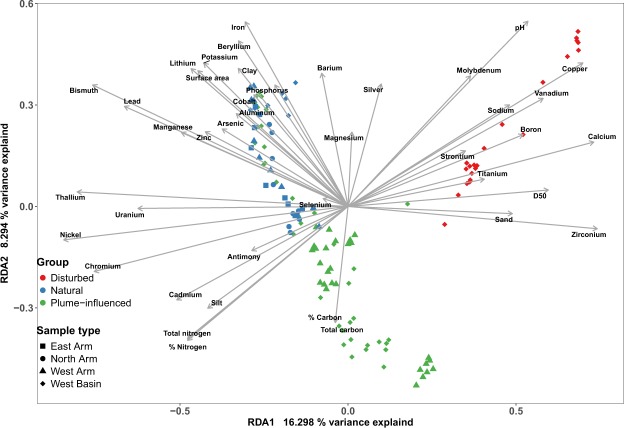
RDA ordination plot of the samples based on weighted Bray-Curtis distances on OTU data and Manhattan distances on metal and metalloid concentrations.

Our results showed a statistically significant and positive correlation (Mantel test with Spearman correlation *ρ* = 0.5, *p* value < 0.01) of the distances between the samples based on physico-chemical parameters and the distances between the samples based on community composition. Consequently, we proceeded with testing the extent to which the physico-chemical parameters explained the relationship between the samples based on community composition, using redundancy analysis (RDA) ordination (Fig. 6). The RDA captured 51% of the total variance in the system, of which 28% was explained by the constraints that the chemical data had on the way samples partitioned based on community composition (Fig. 6). In other words, more than half of the variance explained by the RDA was attributed to the sediment physico-chemical parameters, meaning that much of the microbial community compositional differences observed in the sediment core samples were coincident with the physical and chemical differences between the deposited tailings and the natural sediments. Here as well we show that both microbiology and physico-chemical profiles play a part in differentiating disturbed and the plume-influenced and natural sites. Samples from the disturbed sites are separated from the rest of the site types along axis 1, with Cu, Na, Ca and V as the metals largely contributing to this separation (Fig. 6) as well as pH levels, total N concentration and particle size (Fig. 6). Overall, we found both a difference in the microbiology and the physico-chemical characteristics of disturbed sites compared to plume-influenced and natural sites, two years post-spill.

## Discussion

The cores from the disturbed sites had a larger median particle diameter than those of the natural and plume-influenced sites. This falls within known parameters of mine process tailings, where particles are mainly classified as silt and sand rather than clay^21,22^. Furthermore, the depth-related increase in particle size measured in the deposited tailings and scoured material is as expected for a large single-time deposition event where larger particles would sink faster and would be found at the bottom and lighter smaller particles would settle more slowly and be found at the top. Whereas within natural sediments, a more uniform pattern of particle distribution with depth is expected due to natural processes of relatively constant sedimentation^21^. Therefore, size particle composition supports our assignment of disturbed sites.

It was apparent that samples collected from disturbed sites partitioned separately from the rest of the samples, based on chemistry. The fact that Cu, Ca, and Na contributed significantly to the separation of the disturbed samples can be explained by the composition of the tailings. Mount Polley mine is characterized by alkalic rock, rich in calcites^23^ accounting for the higher concentrations of Ca and Na in the cores from the disturbed sites. The main ore mineral in tailings from Mount Polley mine is chalcopyrite (CuFeS_2_), although bornite (Cu_5_FeS_4_), covellite (CuS), digenite (Cu_9_S_5_) also occur^24^. Tailings produced at Mount Polley mine were characterized to have a Cu content of 65–1475 mg/kg^3^. Furthermore, a recent publication investigating the tailings material deposited along Hazeltine Creek showed the concentrations of Cu to be 88–1020 mg/kg^25^. Other metals and metalloids measured in this study also had similar concentrations to those reported for tailings from the Mount Polley mine^3^. The differences in N content between the natural and plume-influenced sites versus the disturbed site can be explained by the fact that the former contain mainly sediment from terrestrial and water column biological productivity whereas the tailings are mainly composed of alkalic rock^21,23^. Unlike the pore water pH from natural sites, the tailings pore water had an alkaline pH, probably due to the high calcite and low S content of the tailings^3^. This is in agreement with Byrne *et al*.^25^, which reported pH ranges of 7.8–8.5 for the pore water of tailings deposited along Hazeltine Creek. Therefore, our results show that sediment cores from the disturbed sites have similar physical and chemical characteristics to those of tailings deposited in Hazeltine Creek. The variability in the metals and metalloids content between all of the undisturbed and plume-influenced samples is most likely due to the stochasticity of the burial processes seen in all aquatic environments, as well as spatial variations in particle size composition^6,26^. The clustering of core sections from site 12, which is located on the sill, separating the two parts of the West Arm of the lake, into a unique group is presumed to be a function of the rapid water flow velocity at this shallow site (33 m)^27^, which would alter the sedimentation processes (i.e. more erosion and less accumulation) compared to other sites.

While the metal chemistry of the pore water was not measured in this study, Byrne *et al*.^25^ measured the concentration of several ions in the pore water of the top 20 cm of sediments in areas where tailings were deposited. According to Byrne *et al*.^25^, the pore water within deposited material along Hazeltine Creek had elevated levels of Cu (43–1017 μg/L), Fe (63–3510 μg/L), Mn (18–1468 μg/L) and SO_4_^−2^ (40–60 mg/L), with Cu^+2^, Fe^+2^ and Fe^+3^ being the main ions given the pH levels of the pore water^25^. Byrne *et al*.^25^ contend that the mobilization of metals and the release of SO_4_^−2^ to the pore water is the result of infiltration of O_2_ into the sediment, which drives the weathering and oxidation of chalcopyrite found in the tailings^25^. The West Basin’s water-column is oxygenated all the way to the lake bottom throughout the year, therefore, it is possible that O_2_ diffuses into the pore water of the deposited tailings. This suggests that similar chemical reactions occurring in the terrestrial tailings might occur in the pore water of the tailings deposited at the bottom of Quesnel Lake.

Evidence presented here indicates that the composition of the bacterial communities found in core sections from disturbed sites significantly differed from those found in core sections from natural and plume-influenced sites. Indicator OTUs for the disturbed sites came mainly from the abundant class level groups in those sites; they also reflected the chemistry of the pore water as described by Byrne *et al*.^25^. Several of the indicator species for the disturbed sites are known or putative sulfate/sulfur reducers (SRBs). These include Deltaproteobacteria OTU 2000 (*Geobacter*) and OTU 5429 (*Desulfomicrobium*), OTU 3365 (*Fusibacter*), OTU 169 and OTU 522 (Burkholderiaceae), OTU 288 (Rhodocyclaceae). Members of the genus *Desulfomicrobium* are known SO_4_^−2^ reducers found in many environments, including the tailings of copper mines and are active at a wide range of pHs from acidic to basic^28–30^. Members of the genus *Geobacter* are known as dissimilatory reducers of metals (including Fe) and SO_4_^−2^, and can grow in environments low in organic C^31–34^. Similarly, members of the families Burkholderiaceae and Rhodocyclaceae were recently reported in a stable isotope probing-based study to have members capable of metal and SO_4_^−2^ reduction^35^. The genus *Fusibacter* contains members capable of reducing thiosulfate and elemental S to S^−2^ ^36,37^. On the other hand, OTUs associated with SO_4_^−2^/S and metal reduction were either undetected or present in relative abundances less than 0.1% in samples from natural and plume-influenced sites. This is in agreement with a recent study that compared microbial communities in the material deposited within Hazeltine Creek with natural sediments from the shore of Quesnel Lake approximately 2 months post-spill^38^. Although Garris *et al*.^38^ have used a different variable region of the 16 s rRNA gene, which prevents direct comparison of the sequences, the authors have identified similar groups as indicators of tailings deposition sites, namely members of the Burkholderiaceae and Rhodocyclaceae families^38^. Furthermore, Garris *et al*.^38^ reported that open reading frames for the dissimilatory sulfite reductase gene were as much as an order of magnitude more abundant in the deposited material than in natural sediments from the shore of Quesnel Lake^38^. Indicator OTUs for the disturbed sites also included a member of the genus *Georgfuchsia* (OTU 207), members of which are tentative Mn cyclers, and are known as Fe(III), Mn(IV), and nitrate reducers^39^. Another indicator OTU was a member of the genus *Algoriphagus* (OTU 4472) with members capable of Mn(II) oxidation^40^.

Parsing the co-occurrence sub-network for the indicators OTUs (Fig. S6b), two groups are apparent, both with SRB OTUs as central nodes. The left-hand side of the network diagram features *Desulfomicrobium-* and *Fusibacter-*related OTUs as having positive co-occurrence patterns with each other. These are connected to the other larger group within the network through an OTU related to members of the Prolixibacteraceae family. Co-occurrence of members of these genera has been reported in the literature previously and it has been suggested that SRB from class Deltaproteobacteria benefit from the metabolic activity of members from the Prolixibacteraceae family and the genus *Fusibacter* both by receiving H_2_ and short-chained C sources from them under SO_4_^−2^ reducing conditions^41^. The other side of the sub-network had *Geobacter* and a member of the family Burkholderiaceae as central vertices. Members of the Burkholderiaceae family were reported to live in association with other SRB, specifically in environments high in metal concentrations^42,43^. Both were in positive association with member of the genus *Methylotenera* (OTU 3386), which is closely related to methanotrophs^44^ - methanotrophy is a process strongly linked to sulfate reduction^45^. The co-occurence of Burkholderiaceae and methanotrophs was also previously reported in the literature^46,47^. Other members of the right-hand side compartment were also found in sulfate-rich environments, in association with SO_4_^−2^ reducers and potentially involved in metal and H redox (i.e. *Erysipelothrix*-related OTU 1, *Algoriphagus*-related OTU 4472, OTUs classified as belonging to the genera *Flavobacterium* and *Arenimonas*, and those classified as belonging to the family Xanthomonadaceae). Members of the genus *Flavobacterium* were also reported to create an anaerobic environment facilitating S reduction by SRB in glacial foreland soils. Members of the genus *Algoriphagus* were reported to share electron transport with SRB via metal oxidation^37,39,40,42,43,48–52^. Taken together, these associations present evidence for a community of chemolithotrophs and their heterotrophic syntrophs whose metabolic interactions involve transfer of electrons through metals and S cycling in a reducing environment. This makes sense in light of the chemical profiles of the cores from the disturbed sites and is consistent with studies looking at the bacterial communities found in the tailings of metals mines^53,54^. Our results also fall in line with studies conducted on contaminated soils impacted by the Aznalcóllar tailings spill. These studies showed persistence of legacy bacterial community several years post spill as well as significant differences in soil enzymatic activity between impacted and unimpacted sites^55,56^.

The dominant classes for the natural and plume-influenced sites (Alphaproteobacteria, Acidobacteria and Actinobacteria) agreed with those reported for subaqueous sediment from the shoreline of Quesnel Lake^38^. While it was not possible to classify the indicator OTUs for the plume-influenced or the undisturbed sites to the genus level, there were several OTUs (OTU 3458, OTU 4047, OTU 10222) which were classified as members of the family Nitrosomonadaceae that contains members that are known as ammonia oxidisers^57^. Another OTU (OTU 3486) was classified as a member of the family Xanthobacteraceae, whose members are also involved in the N cycle via N fixation^58^. Finding indicator members of the community that are mainly involved in N cycling makes sense in light of the high content of N found in the natural and plume-influenced sites. These findings are also in agreement with Garris *et al*.^38^, which reported high levels of open reading frames (ORFs) for genes associated with the N cycle in the Quesnel Lake shoreline subaqueous sediments.

Lastly, our data indicate that only one of the top sections from the plume-influenced sites clustered with the disturbed sites and that none of the top sections from the disturbed sites clustered with the undisturbed or plume-influenced sites. This could be explained by the sedimentation patterns in Quesnel Lake, which has three major inputs of turbidity: Niagara Creek in the East Arm, Mitchell River in the North Arm, and Horsefly River in the West Arm^59^. However, even in the Horsefly River delta the sedimentation rate was estimated to be 0.35 to 0.72 mm/y, while the sedimentation rate in the West Basin which has no major input of sediment is 0.22 mm/y^5^. This might explain why little natural sediment cover, as inferred from the surface C and N content, has accumulated at the deposition sites two years post spill. The West Basin also has a relatively short hydraulic residence time of ~12 weeks^60,61^, enabling the discharge of plume materials from the West Basin to the Quesnel River in the fall of 2014 and limiting the settling of the plume materials until winter ice formed. Eastward of the sill, the plume from the spill event might have mixed with natural sediment coming from the Horsefly River.

The stark differences observed between the microbial communities in the deposited tailings and those at the non-impacted, natural sites highlights the need for continued monitoring of the lake biota, including the below sediment–water interface microbiome, as well as benthic, planktonic and higher food chain species, to assess the impact of the tailings deposition on the whole lake ecosystem. For instance, biochemical sulphur cycling can impact metal geochemistry, with SRB facilitating formation of low solubility metal suphides and metal immobilization, and sulphur oxidizing microorganisms accelerating oxidation of metal sulphides to solubilize metals^19,62^. The redox state of metals can influence their bioavailability to aquatic organisms^2^. These potential impacts on lake geochemistry are of extreme importance given that Quesnel Lake has historically accounted for over 30% of the anadromous sockeye salmon (*Oncorhynchus nerka*) runs in the Fraser River^63^, and that the lake also supports a resident population of kokanee salmon (*Oncorhynchus nerka*), which serve as the main prey for rainbow trout (*Oncorhynchus mykiss*)^60^, making Quesnel Lake economically important both for commercial and recreational fishing. The current study, however, does not provide any concrete information on the actual flux of S or metal cycling in the deposited tailings, but suggests that this be measured and monitored in future work.

## Conclusions

Two years after a catastrophic tailings dam breach and the deposition of tailings and scoured material into Quesnel Lake, there are clear differences in the bacterial communities within the top 10 cm sediment layer of the main deposition area sites compared to those within the top 10 cm layer of sites outside the main deposition zone. The main bacterial indicators of these differences appear to be legacy bacteria indicating a metabolic potential required to inhabit tailings (i.e. mainly involving SO_4_^−2^ and metals reduction), which was not the case for the natural sites. Given the key role sediment bacteria play in lacustrine ecosystems, our results indicate a potential impact of the spill on biogeochemical cycles of the West Basin of Quesnel Lake in particular, which could be long lasting given the slow sedimentation rate in that basin, however further research is needed in order to confirm that.

## Methods and Materials

Detailed materials and methodology are found in on-line Supplementary Material.

### Sample collection and processing

Samples were collected from all basins of Quesnel Lake (Fig. 1) a long (~100 km east to west span), narrow (~2.7 km mean width), and deep (maximum depth of 511 m and mean depth of 157 m) lake with a surface area of 266 km^2^ and water volume of 41.8 km^3,60^. It is considered relatively pristine and oligotrophic, with an historic mean total P concentration of 2.0 μg/L and total dissolved solids ranging between 60 mg/L and 90 mg/L^64,65^. Fourteen sites were sampled: seven sites from within the West Basin; five additional sites from within the West Arm eastwards of the sill; and one each from within the North and East Arms (Fig. 1). Latitude and longitude site coordinates are specified in Table S2. With the exception of site 16 for which the core retrieved was only 7 cm in length, for each site a ~50 cm sediment core was collected and kept at *in-situ* temperature for up to 8 h. The line drawing for Fig. 1 and embedded DigitalGlobe image were compiled with Adobe® Illustrator® Vs. 10. Satellite image was adopted from Google Earth Pro.

For each of the cores, the top 10 cm was sectioned into 1 cm intervals using a clean, sterile spatula. Each core section was divided in half and the halves stored in separate sterile Whirl-Pack® bags (Sigma-Aldrich, ON, Canada) at −20 °C. Samples for microbiological analyses were shipped frozen on dry ice to the University of British Columbia (Vancouver, BC, Canada). Samples intended for physical and chemical analysis were shipped frozen to the University of Northern British Columbia (Prince George, BC, Canada).

Water samples were collected from the sediment–water interface in each core as soon as the core was removed from the corer. The sampling was done using a syringe which was kept in the dark at *in-situ* temperature until analysis for phosphate, nitrate, nitrite, ammonia, and Fe concentrations were performed up to 8 h from time of collection, at the Quesnel River Research Centre (Likely, BC, Canada).

### Chemical analysis

For the measurement of metal content and pH of pore-water, samples were homogenized and dried, before being shipped to ALS Environmental Laboratory (Burnaby, BC, Canada). Carbon and nitrogen analysis for the sediment core samples was performed using a Costech Elemental Combustion System (ECS 4010) at UNBC.

Particle size composition (including D_50_, %clay, %silt and %sand) was determined at the QRRC using a Malvern Mastersizer 3000 laser particle sizer. Water chemistry was done on water filtered through 0.22-µm pore syringe filter, using Chemetrics (CHEMetrics inc, Midland, VA, USA) Chemets kits for ammonia (K-1413), phosphate (K-8513), nitrate (K-6904), and iron (K-6210).

Further details can be found at the supplementary Method and Materials.

### DNA extraction and sequencing

DNA from each of the sections was extracted using the fast DNA^TM^ spin kit for soil according to the manufacturer’s instructions (MP bio Solon, OH, USA). Amplicons for the v4 variable region of the 16 S rRNA gene were produced from target DNA by polymerase chain reaction (PCR) with primers: 515 f 5′ GTGCCAGCMGCCGCGGTAA 3′, 806r 5′ GGACTACHVGGGTWTCTAAT 3′ using methods described previously^66^. Amplicons were sequenced using Illumina MiSeq technology by microbiome INSIGHTS (Vancouver, BC, Canada).

### Processing of raw sequences

All pre-processing and sequence quality control steps were performed using USEARCH (v 9.0.2132) according to the UPARSE pipeline for paired end reads (http://drive5.com/usearch/manual/uparse_pipeline.html; accessed 1 Oct 2016)^67^. Bacterial OTUs were classified to phylum, class, family, and genus level using the USEARCH utax classifier with the RDP trainset 14 (RDP; http://rdp.cme.msu.edu/) as taxonomic reference set. Following classification, sequences not classified as bacteria (i.e. unclassified to the domain level, Eukaryotes, Archaea, or organelles) were removed from the dataset.

Following denoising and quality filtration, samples with fewer than 1000 sequences were removed from the analysis to guarantee decent coverage of bacterial OTUs.

This resulted in 129 samples that were selected for further analysis (Table S2).

Demultiplexed raw sequence files were submitted to the National Center for Biotechnology Information Sequence Read Archive under SRA Accession no under bioproject PRJNA482848, SRA accession SRP155267 individual sample accession numbers can be found in Table S2.

### Statistics and community analysis

All community and statistical analyses were performed in R (version 3.4.3), a detailed description of methods used is found in the Supplementary Methods and Materials. In brief, distances between the composition of the communities was calculated using weighted Bray-Curtis dissimilarity index and visualized using non-metric multidimensional scaling (NMDS). K-means and Unweighted Pair Group Method with Arithmetic Mean (UPGMA) clustering were used to group the samples into clusters. Supervised learning with Random forest was used to find indicator OTUs for each of the sample types. Redundancy Analysis (RDA) was used to test how well the chemical and physical attributes of the sample explain the distances between the bacterial communities from each of the samples. Where relevant, statistical tests were considered significant for *p* < 0.05.

## Supplementary information

Supplementary info

## Data Availability

All data generated or analyzed during this study are included in this article and its Supplementary Information files or in public repositories indicated in the methods section.
