# Supplementary material for: The bacterial community of Quesnel Lake sediments impacted by a catastrophic mine tailings spill differ in composition from those at undisturbed locations – two years post-spill
Source: Sci Rep. 2019 Feb 25;9:2705. doi: 10.1038/s41598-019-38909-9 (PMC6389986; doi:10.1038/s41598-019-38909-9)
Supplement: Supplementary file 1 — Supplementary info [file 41598_2019_38909_MOESM1_ESM.pdf]

Manuscript in preparation for publication in Scientific Reports

Running title: Lasting impact of spill on bacterial community from lake sediment

Title: The bacterial community of Quesnel Lake sediments impacted by a catastrophic mine tailings spill differ in composition from those at undisturbed locations – two years post-spill

Authors: Hatam I[1*], Petticrew EL[2], French TD[2, 3], Owens PN[3], Laval B[4], Baldwin SA[1*]

[1]Department of Chemical and Biological Engineering, University of British Columbia, Vancouver, British Columbia, V6T1Z3, Canada

[2]Geography Program and Quesnel River Research Centre, University of Northern British Columbia, Prince George, British Columbia, V2N4Z9, Canada

[3]Environmental Science Program and Quesnel River Research Centre, University of Northern British Columbia, Prince George, British Columbia, V2N4Z9, Canada

[4] Department of Civil Engineering, University of British Columbia Vancouver, British Columbia, V6T1Z3, Canada

*Corresponding Authors: idohatam@gmail.com, sue.baldwin@ubc.ca

Competing interests

The authors declare that they have no competing interests, both financial and non-financial.

Manuscript in preparation for publication in Scientific Reports

Running title: Lasting impact of spill on bacterial community from lake sediment

Title: The bacterial community of Quesnel Lake sediments impacted by a catastrophic mine tailings spill differ in composition from those at undisturbed locations – two years post-spill

Authors: Hatam I[1*], Petticrew EL[2], French TD[2, 3], Owens PN[3], Laval B[4], Baldwin SA[1*]

[1]Department of Chemical and Biological Engineering, University of British Columbia, Vancouver, British Columbia, V6T1Z3, Canada

[2]Geography Program and Quesnel River Research Centre, University of Northern British Columbia, Prince George, British Columbia, V2N4Z9, Canada

[3]Environmental Science Program and Quesnel River Research Centre, University of Northern British Columbia, Prince George, British Columbia, V2N4Z9, Canada

[4] Department of Civil Engineering, University of British Columbia Vancouver, British Columbia, V6T1Z3, Canada

*Corresponding Authors: idohatam@gmail.com, sue.baldwin@ubc.ca

Competing interests

The authors declare that they have no competing interests, both financial and non-financial.

Supplementary Methods and Materials

Sample collection and processing

Samples were collected from all basins of Quesnel Lake (Figure 1). A total of 14 sites were sampled: seven sites from within the West Basin; an additional five sites from within the West Arm east of the sill; and one each from within the North and East Arms (Figure 1). Latitude and longitude site coordinates are provided in Supplementary Table 2. For each site a ~ 50 cm sediment core was collected, using a Slo-corer with 10 cm diameter core barrels (BDR Research Limited, East Dover, NS, Canada)[1]. The importance of using the Slo-corer is that it maintains the sediment–water interface, unlike most other corers (e.g. gravity-corers) and grab samplers (e.g. Ekman grab). Water depth in which each sample was collected along with the total length of each core, is specified in Supplementary Table 2.

Cores were kept at *in-situ* temperature for up to 8 h on the boat and were brought back to the field laboratory at the Quesnel River Research Center (QRRC, Likely, BC, Canada).

For chemical and microbiological data, we focused on the top 10 cm of the sediment core. For each of the cores destined for chemical and microbiological analysis, the top 10 cm was sectioned into 1 cm intervals using a clean, sterile spatula. Each core section was divided in half and the halves stored in separate sterile Whirl-Pack® bags (Sigma-Aldrich, ON, Canada) at -20℃ until shipment to the processing laboratory. Samples intended for microbiological analysis were shipped frozen on dry ice to the University of British Columbia (Vancouver, BC, Canada). Samples intended for metal analysis were shipped frozen to the University of Northern British Columbia (UNBC,

Prince George, BC, Canada).

Water at the sediment–water interface were collected on site using a syringe from the water at the top of the core-barrel; this was done carefully and without resuspending sediment particles at the top of the core. The water was kept dark and at *in-situ* temperature, and samples were analyzed for phosphate, nitrate, nitrite, ammonia, iron, and sulfate upon arrival to the QRRC.

Physical and chemical analysis

For the measurement of metal content and pH of pore-water, samples were homogenized and dried, before being shipped to ALS Environmental Laboratory (Burnaby, BC, Canada), where metals content of sediment was measured by drying the samples at <60 ^{o}C overnight and sieving using a non-metallic <200 μm sieve. Samples were then analyzed for trace, minor and major elements using aqua regia digestion followed by ICP-MS and ICP-AES at ALS and results represent the leachable portion of the trace elements associated with the sediment,

Water chemistry was done on water filtered through 0.22 µm pore syringe filter, using Chemetrics (CHEMetrics inc, Midland, VA, USA) Chemets kits for ammonia (K-1413), phosphate (K-8513), nitrate (K-6904), and iron (K-6210). Water pH was measured using pH strips.

A Costech 4010 CHNSO analyzer was used to combust the samples via the standard sequential combustion/reduction setup recommended by Costech (see Costech manual) with a flowrate of 100 mL/min of helium 5.0 as the carrier gas. The combustion oven was set to 1000°C, the reduction oven to 650°C, and the GC oven to

45°C. Gases were then separated on a standard 2 m (Costech 051081) column and quantified with the built-in thermal conductivity detector (TCD).

Particle size composition (including D50, %clay, %silt and %sand) was determined at the QRRC using a Malvern Mastersizer 3000 laser particle sizer after pretreatment with hydrogen peroxide and chemical (sodium hexametaphosphate) and ultrasonic dispersion.

DNA extraction and sequencing

DNA from each of the sections was extracted using the fast DNA™ spin kit for soil according to the manufacturer's instructions (MP bio Solon, OH, USA). Amplicons for the v4 variable region of the 16S rRNA gene were produced from target DNA by polymerase chain reaction (PCR) with primers: 515f 5' GTGCCAGCMGCCGCGGTAA 3', 806r 5' GGACTACHVGGGTWTCTAAT 3' using methods described previously[2]. Amplicons were sequenced using Illumina MiSeq technology by microbiome INSIGHTS (Vancouver, BC, Canada).

Processing of raw sequences

All pre-processing and sequence quality control steps were performed using USEARCH (v 9.0.2132, 32 bit for Linux) according to the UPARSE pipeline (http://drive5.com/usearch/manual/uparse_pipeline.html; date accessed 1 Oct 2016)[3]. Briefly, paired end reads were combined, as suggested by the UPARSE pipeline and unpaired reads or reads with a maximum expected error probability > 1 were removed from the analysis. As suggested by Huse et al., the remaining sequences were pre-clustered using the furthest neighbor algorithm with 1% sequence dissimilarity as the threshold to prevent over estimation of operational taxonomic units (OTUs) richness,

using the cluster smallmem command[4]. OTUs were assigned using the UPARSE greedy algorithm for an OTU definition of 97% sequence similarity. Although this step also removes chimeras, a dedicated chimera removal step was additionally used post OTU clustering using the UCHIME 2 algorithm with SILVA gold dataset as reference[5]. Global singleton OTUs (those that are represented by a single sequence in the entire dataset) were removed due to their unknown nature. The bacterial OTUs were classified to phylum, class, family, and genus level using the USEARCH utax classifier with the RDP trainset 14 (RDP; http://rdp.cme.msu.edu/) as taxonomic reference set, confidence threshold was set at 60% (RDP; http://rdp.cme.msu.edu/). Following classification, sequences not classified as bacteria (i.e. unclassified to the domain level, Eukaryotes, Archaea, or organelles) were removed from the dataset. OTUs removed from the dataset accounted for an avarage of 84 seqs/ sample and an average of 14 OTUs/ sample, with maximum values of 300 and 25 respectively.

Demultiplexed raw sequence files were submitted to the National Center for Biotechnology Information Sequence Read Archive under SRA Accession no under bioproject PRJNA482848, SRA accession SRP155267 individual sample accession numbers can be found in Table S2.

Statistics and community analysis

All community and statistical analyses were performed in R (version 3.4.3). Samples were subsampled to the same total number of reads (1870) using the vegan package (version 2.4-1) rrerafy function, to eliminate coverage-dependent biases to alpha diversity estimations[6, 7]. Expected number of OTUs was estimated using the nonparametric

richness estimator Chao 1[8]. Library coverage was estimated by calculating the ratio of observed to expected OTU numbers (observed OTUs/Chao1). Coverage for all samples was ~50% of the expected OTU richness, which indicates adequate sequencing depth (Supplementary Table 2).

Differences between the compositions of the communities (beta diversity) in each of the samples were performed using the metaMDS function from the vegan package with weighted Bray-Curtis dissimilarity index[7, 9]. In order to prevent biases of the beta diversity estimation due to highly abundant OTUs, the samples were log10 transformed prior to the generation of the distance matrix[10]. Non-metric multidimensional scaling (NMDS) ordination was used to visualize the distances between the communities based on the matrix generated with the metaMDS. K-means clustering along with UPGMA on the Bray-Curtis distances were used to assess the number of clusters to which samples group (Supplementary Figures 5 and 7), and the non-parametric Analysis of Molecular Variance (AMOVA) was used to test for the statistical significance of the grouping[11, 12] using the ADONIS function from the vegan package[7]. Indicator OTUs for the different groups were determined using the R randomForest package (version 4.6-12)[13]. For this step, only OTUs that appeared in 15% of the samples were considered in order to reduce noise, and OTUs were normalized using relative abundance (i.e. proportion from the population/sample). This analysis then assigns an indicator value (mean decrease accuracy) to all the OTUs. Consequently, we used a broken stick model on the mean decrease accuracy value to select those with significant contribution as indicators.

Association network analysis on bacterial communities from disturbed sites was done using R’s netassoc package (version 0.6.3) with default settings (rho >0.5, $p<0.05$)[14].

Prior to the analysis, OTUs not represented in at least 16 samples, as well as not accounting for at least 0.025% of the sequences in a single sample and not accounting for 0.1% of the total sequences, were removed to reduce noise[15].

The network was visualized and analyzed using the tidygraph and ggraph packages.

Grouping based on chemistry data was done with Principal Component Analysis (PCA) using vegan's RDA command[7], with Euclidean distances on log transformed metal concentrations. The number of clusters was evaluated similarly to the community data (Supplementary Figures 2 and 3).

Correlation between the distances based on chemistry and community data were tested using a Mantel test with 999 permutations on Spearman correlation coefficient. The significance of the clustering based on chemical profiles was tested with Multi Response Permutation Procedure (MRPP), using vegan's mrpp function with default settings[7, 16].

The constraining effect of the chemistry on the distances between the bacterial communities was tested using Redundancy Analysis (RDA) using vegan's RDA function[7] on log transformed chemical data and Hellinger transformed OTU abundances. All statistical tests were considered significant for p value < 0.05, and where appropriate the p value was adjusted with Bonferroni's correction.

References

1. Milligan, T. G. & Law, B. A. Contaminants at the sediment-water interface: implications for environmental impact assessment and effects monitoring. *Environ Sci Technol* **47**, 5828-5834 (2013).

2. Caporaso, J. G. *et al*. Ultra-high-throughput microbial community analysis on the Illumina HiSeq and MiSeq platforms. *ISME J* **6**, 1621 (2012).

3. Edgar, R. UPARSE: highly accurate OTU sequences from microbial amplicon reads. *Nat. Methods* **10**, 996-998 (2013).

4. Huse, S. M., Welch, D. M., Morrison, H. G. & Sogin, M. L. Ironing out the wrinkles in the rare biosphere through improved OTU clustering. *Environ Microbiol* **12**, 1889-1898 (2010).

5. Edgar, R. UCHIME2: improved chimera prediction for amplicon sequencing. *bioRxiv* (2016).

6. Gihring, T. M., Green, S. J. & Schadt, C. W. Massively parallel rRNA gene sequencing exacerbates the potential for biased community diversity comparisons due to variable library sizes. *Environ Microbiol* **14**, 285-290 (2012).

7. Oksanen, J. *et al*. vegan: Community Ecology Package. R package version 2.4-1. 2016. (2016).

8. Chao, A. & Shen, T. Nonparametric estimation of Shannon's index of diversity when there are unseen species in sample. *Environ and Ecol Stat* **10**, 429-443 (2003).

9. Bray, J. R. & Curtis, J. T. An Ordination of the Upland Forest Communities of Southern Wisconsin. *Ecol Monograph* **27**, 325-349 (1957).

10. Costa, P. S. *et al*. Metagenome of a Microbial Community Inhabiting a Metal-Rich Tropical Stream Sediment. *PLOS ONE* **10**, e0119465 (2015).

11. Excoffier, L., Smouse, P. E. & Quattro, J. M. Analysis of molecular variance inferred from metric distances among DNA haplotypes: application to human mitochondrial DNA restriction data. *Genetics* **131**, 479-491 (1992).

12. Martin, A. P. Phylogenetic approaches for describing and comparing the diversity of microbial communities. *Appl Environ Microbiol* **68**, 3673-3682 (2002).

13. Liaw, A. & Wiener, M. Classification and regression by randomForest. *R News* **2**, 18-22 (2002).

14. Morueta-Holme, N. *et al*. A network approach for inferring species associations from co-occurrence data. *Ecography* **39**, 1139-1150 (2016).

15. Faust, K. & Raes, J. Microbial interactions: from networks to models. *Nat Rev Microbiol* **10**, 538 (2012).

16. Van Sickle, J. Using mean similarity dendrograms to evaluate classifications. * J Agric Biol Environ Stat* **2**, 370-388 (1997).

Table S1 Chemistry of water at the water sediment interface of selected sites

Site	pH	NO_3^-/NO_2^- N	NH_3	PO_4	Fe(diss)
QL1	7	0.25	0	0	0
QL8	7	0	0	0	0
QL4	7	0	0	0	0
QL3	7	0.15/0.15	0	0	0
QL5	7	0	0	0	0
QL6	7	0/0.1	0	0	0

Table S2 SRA accession numbers collection dates coordinates and richness measurements for each sample

Site	*Sample*	*Sample name on SRA*	*SRA accession*	*Collection date*	*latitude and longtitude*	*Obs.OTUs*	*Chao1*	*% Coverage*
QL1	QL1s1	QL25s1	SRS3596409	2016-07-11	52.5725 N 121.54167 W	483	1052	46
QL1	QL1s2	QL25s2	SRS3596411	2016-07-11	52.5725 N 121.54167 W	474	1130	42
QL1	QL1s3	QL25s3	SRS3596373	2016-07-11	52.5725 N 121.54167 W	458	1081	42
QL1	QL1s4	QL25s4	SRS3596374	2016-07-11	52.5725 N 121.54167 W	471	1068	44
QL1	QL1s5	QL25s5	SRS3596375	2016-07-11	52.5725 N 121.54167 W	407	862	47
QL1	QL1s6	QL25s6	SRS3596415	2016-07-11	52.5725 N 121.54167 W	406	894	45
QL1	QL1s7	QL25s7	SRS3596447	2016-07-11	52.5725 N 121.54167 W	402	847	47
QL1	QL1s8	QL25s8	SRS3596408	2016-07-11	52.5725 N 121.54167 W	400	868	46
QL1	QL1s9	QL25s9	SRS3596487	2016-07-11	52.5725 N 121.54167 W	444	920	48
QL1	QL1s10	QL25s10	SRS3596410	2016-07-11	52.5725 N 121.54167 W	418	840	50

QL8	QL8s1	QL27s1	SRS3596430	2016-07-11	52.50889 N 121.51083 W	448	1008	44
QL8	QL8s2	QL27s2	SRS3596427	2016-07-11	52.50889 N 121.51083 W	451	1036	44
QL8	QL8s3	QL27s3	SRS3596428	2016-07-11	52.50889 N 121.51083 W	471	1139	41
QL8	QL8s4	QL27s4	SRS3596432	2016-07-11	52.50889 N 121.51083 W	433	995	43
QL8	QL8s5	QL27s5	SRS3596434	2016-07-11	52.50889 N 121.51083 W	465	1052	44
QL8	QL8s6	QL27s6	SRS3596431	2016-07-11	52.50889 N 121.51083 W	458	965	47
QL8	QL8s7	QL27s7	SRS3596433	2016-07-11	52.50889 N 121.51083 W	466	922	51
QL8	QL8s8	QL27s8	SRS3596435	2016-07-11	52.50889 N 121.51083 W	511	1109	46
QL8	QL8s9	QL27s9	SRS3596436	2016-07-11	52.50889 N 121.51083 W	480	978	49
QL8	QL8s10	QL27s10	SRS3596429	2016-07-11	52.50889 N	462	1003	46

					121.51083 W			
QL3	QL3s1	QL31s1	SRS3596467	2016-07-12	52.54722 N 121.52833 W	471	1008	47
QL3	QL3s2	QL31s2	SRS3596465	2016-07-12	52.54722 N 121.52833 W	374	747	50
QL3	QL3s3	QL31s3	SRS3596464	2016-07-12	52.54722 N 121.52833 W	359	707	51
QL3	QL3s4	QL31s4	SRS3596463	2016-07-12	52.54722 N 121.52833 W	358	762	47
QL3	QL3s5	QL31s5	SRS3596462	2016-07-12	52.54722 N 121.52833 W	341	694	49
QL3	QL3s6	QL31s6	SRS3596461	2016-07-12	52.54722 N 121.52833 W	313	588	53
QL3	QL3s7	QL31s7	SRS3596460	2016-07-12	52.54722 N 121.52833 W	360	660	55
QL3	QL3s8	QL31s8	SRS3596459	2016-07-12	52.54722 N 121.52833 W	342	659	52
QL3	QL3s9	QL31s9	SRS3596458	2016-07-12	52.54722 N 121.52833 W	321	580	55

QL3	QL3s10	QL31s10	SRS3596468	2016-07-12	52.54722 N 121.52833 W	329	620	53
QL5	QL5s1	QL32s1	SRS3596480	2016-07-12	52.52806 N 121.51778 W	472	1087	43
QL5	QL5s2	QL32s2	SRS3596481	2016-07-12	52.52806 N 121.51778 W	480	1082	44
QL5	QL5s3	QL32s3	SRS3596482	2016-07-12	52.52806 N 121.51778 W	412	906	46
QL5	QL5s4	QL32s4	SRS3596501	2016-07-12	52.52806 N 121.51778 W	414	918	45
QL5	QL5s5	QL32s5	SRS3596483	2016-07-12	52.52806 N 121.51778 W	447	979	46
QL5	QL5s6	QL32s6	SRS3596484	2016-07-12	52.52806 N 121.51778 W	450	921	49
QL5	QL5s7	QL32s7	SRS3596485	2016-07-12	52.52806 N 121.51778 W	432	921	47
QL5	QL5s9	QL32s9	SRS3596486	2016-07-12	52.52806 N 121.51778 W	419	928	45

QL6	QL6s1	QL34s1	SRS3596479	2016-07-12	52.51917 N 121.51 W	447	1072	42
QL6	QL6s2	QL34s2	SRS3596495	2016-07-12	52.51917 N 121.51 W	450	1086	41
QL6	QL6s3	QL34s3	SRS3596494	2016-07-12	52.51917 N 121.51 W	390	901	43
QL6	QL6s4	QL34s4	SRS3596497	2016-07-12	52.51917 N 121.51 W	398	919	43
QL6	QL6s5	QL34s5	SRS3596496	2016-07-12	52.51917 N 121.51 W	410	956	43
QL6	QL6s6	QL34s6	SRS3596491	2016-07-12	52.51917 N 121.51 W	416	895	47
QL6	QL6s7	QL34s7	SRS3596490	2016-07-12	52.51917 N 121.51 W	407	888	46
QL6	QL6s8	QL34s8	SRS3596493	2016-07-12	52.51917 N 121.51 W	398	854	47
QL6	QL6s9	QL34s9	SRS3596492	2016-07-12	52.51917 N 121.51 W	403	904	45
QL6	QL6s10	QL34s10	SRS3596478	2016-07-12	52.51917 N 121.51 W	393	900	44
QL9	QL9s2	QL36s2	SRS3596488	2016-07-14	52.50367 N 121.49242 W	430	924	46
QL9	QL9s3	QL36s3	SRS3596385	2016-07-14	52.50367 N	404	883	46

					121.49242 W			
QL9	QL9s4	QL36s4	SRS3596386	2016-07-14	52.50367 N 121.49242 W	335	713	47
QL9	QL9s5	QL36s5	SRS3596383	2016-07-14	52.50367 N 121.49242 W	266	596	45
QL9	QL9s6	QL36s6	SRS3596384	2016-07-14	52.50367 N 121.49242 W	213	473	45
QL9	QL9s7	QL36s7	SRS3596381	2016-07-14	52.50367 N 121.49242 W	239	522	46
QL9	QL9s8	QL36s8	SRS3596382	2016-07-14	52.50367 N 121.49242 W	209	472	44
QL9	QL9s9	QL36s9	SRS3596379	2016-07-14	52.50367 N 121.49242 W	202	435	46
QL9	QL9s10	QL36s10	SRS3596489	2016-07-14	52.50367 N 121.49242 W	240	527	45
QL11	QL11s3	QL38s3	SRS3596377	2016-07-14	52.49278 N 121.43494 W	408	795	51
QL11	QL11s4	QL38s4	SRS3596378	2016-07-14	52.49278 N 121.43494 W	355	641	55

QL11	QL11s5	QL38s5	SRS3596392	2016-07-14	52.49278 N 121.43494 W	371	827	45
QL11	QL11s6	QL38s6	SRS3596391	2016-07-14	52.49278 N 121.43494 W	379	826	46
QL11	QL11s7	QL38s7	SRS3596390	2016-07-14	52.49278 N 121.43494 W	348	607	57
QL11	QL11s8	QL38s8	SRS3596389	2016-07-14	52.49278 N 121.43494 W	299	557	54
QL11	QL11s9	QL38s9	SRS3596396	2016-07-14	52.49278 N 121.43494 W	345	617	56
QL11	QL11s10	QL38s10	SRS3596380	2016-07-14	52.49278 N 121.43494 W	342	612	56
QL13	QL13s1	QL40s1	SRS3596394	2016-07-15	52.47531 N 121.35831 W	460	1020	45
QL13	QL13s2	QL40s2	SRS3596393	2016-07-15	52.47531 N 121.35831 W	454	1000	45
QL13	QL13s3	QL40s3	SRS3596388	2016-07-15	52.47531 N 121.35831 W	442	1010	44
QL13	QL13s4	QL40s4	SRS3596387	2016-07-15	52.47531 N	422	936	45

					121.35831 W			
QL13	QL13s5	QL40s5	SRS3596401	2016-07-15	52.47531 N 121.35831 W	431	882	49
QL13	QL13s6	QL40s6	SRS3596402	2016-07-15	52.47531 N 121.35831 W	436	1006	43
QL13	QL13s8	QL40s8	SRS3596405	2016-07-15	52.47531 N 121.35831 W	394	853	46
QL13	QL13s10	QL40s10	SRS3596395	2016-07-15	52.47531 N 121.35831 W	412	864	48
QL14	QL14s1	QL43s1	SRS3596397	2016-07-15	52.48867 N 121.34358 W	432	939	46
QL14	QL14s2	QL43s2	SRS3596398	2016-07-15	52.48867 N 121.34358 W	444	951	47
QL14	QL14s3	QL43s3	SRS3596399	2016-07-15	52.48867 N 121.34358 W	404	869	46
QL14	QL14s4	QL43s4	SRS3596400	2016-07-15	52.48867 N 121.34358 W	439	1036	42
QL14	QL14s5	QL43s5	SRS3596403	2016-07-15	52.48867 N 121.34358 W	407	741	55

QL14	QL14s6	QL43s6	SRS3596406	2016-07-15	52.48867 N 121.34358 W	434	910	48
QL14	QL14s7	QL43s7	SRS3596451	2016-07-15	52.48867 N 121.34358 W	434	911	48
QL14	QL14s8	QL43s8	SRS3596450	2016-07-15	52.48867 N 121.34358 W	486	1134	43
QL14	QL14s9	QL43s9	SRS3596453	2016-07-15	52.48867 N 121.34358 W	461	1090	42
QL14	QL14s10	QL43s10	SRS3596404	2016-07-15	52.48867 N 121.34358 W	434	954	46
QL12	QL12s1	QL44s1	SRS3596455	2016-07-15	52.48902 N 121.36831 W	462	998	46
QL12	QL12s2	QL44s2	SRS3596454	2016-07-15	52.48902 N 121.36831 W	469	1041	45
QL12	QL12s3	QL44s3	SRS3596457	2016-07-15	52.48902 N 121.36831 W	483	1034	47
QL12	QL12s4	QL44s4	SRS3596456	2016-07-15	52.48902 N 121.36831 W	529	1265	42
QL12	QL12s5	QL44s5	SRS3596449	2016-07-15	52.48902 N	507	1088	47

					121.36831 W			
QL12	QL12s6	QL44s6	SRS3596448	2016-07-15	52.48902 N 121.36831 W	507	1138	45
QL12	QL12s7	QL44s7	SRS3596472	2016-07-15	52.48902 N 121.36831 W	522	1170	45
QL12	QL12s8	QL44s8	SRS3596473	2016-07-15	52.48902 N 121.36831 W	536	1231	44
QL12	QL12s9	QL44s9	SRS3596470	2016-07-15	52.48902 N 121.36831 W	532	1098	48
QL12	QL12s10	QL44s10	SRS3596452	2016-07-15	52.48902 N 121.36831 W	528	1177	45
QL16	QL16s1	QL46s1	SRS3596471	2016-07-16	52.51358 N 121.23156 W	382	822	47
QL16	QL16s2	QL46s2	SRS3596476	2016-07-16	52.51358 N 121.23156 W	413	918	45
QL16	QL16s3	QL46s3	SRS3596477	2016-07-16	52.51358 N 121.23156 W	424	902	47
QL16	QL16s4	QL46s4	SRS3596474	2016-07-16	52.51358 N 121.23156 W	351	716	49

QL16	QL16s5	QL46s5	SRS3596475	2016-07-16	52.51358 N 121.23156 W	371	766	48
QL16	QL16s6	QL46s6	SRS3596466	2016-07-16	52.51358 N 121.23156 W	328	606	54
QL16	QL16s7	QL46s7	SRS3596469	2016-07-16	52.51358 N 121.23156 W	312	605	52
QL18	QL18s2	QL48s2	SRS3596438	2016-07-16	52.58286 N 121.02828 W	400	895	45
QL18	QL18s3	QL48s3	SRS3596439	2016-07-16	52.58286 N 121.02828 W	427	892	48
QL18	QL18s4	QL48s4	SRS3596437	2016-07-16	52.58286 N 121.02828 W	416	893	47
QL18	QL18s5	QL48s5	SRS3596444	2016-07-16	52.58286 N 121.02828 W	404	870	46
QL18	QL18s6	QL48s6	SRS3596443	2016-07-16	52.58286 N 121.02828 W	364	751	48
QL18	QL18s7	QL48s7	SRS3596442	2016-07-16	52.58286 N 121.02828 W	370	699	53
QL18	QL18s8	QL48s8	SRS3596441	2016-07-16	52.58286 N	387	741	52

					121.02828 W			
QL18	QL18s9	QL48s9	SRS3596446	2016-07-16	52.58286 N 121.02828 W	375	718	52
QL18	QL18s10	QL48s10	SRS3596440	2016-07-16	52.58286 N 121.02828 W	372	743	50
QL17	QL17s1	QL49s1	SRS3596421	2016-07-16	52.51022 N 121.01133 W	428	874	49
QL17	QL17s2	QL49s2	SRS3596422	2016-07-16	52.51022 N 121.01133 W	413	746	55
QL17	QL17s3	QL49s3	SRS3596423	2016-07-16	52.51022 N 121.01133 W	428	841	51
QL17	QL17s4	QL49s4	SRS3596424	2016-07-16	52.51022 N 121.01133 W	436	895	49
QL17	QL17s5	QL49s5	SRS3596417	2016-07-16	52.51022 N 121.01133 W	390	804	48
QL17	QL17s6	QL49s6	SRS3596418	2016-07-16	52.51022 N 121.01133 W	420	905	46
QL17	QL17s7	QL49s7	SRS3596419	2016-07-16	52.51022 N 121.01133 W	395	730	54

QL17	QL17s8	QL49s8	SRS3596420	2016-07-16	52.51022 N 121.01133 W	397	750	53
QL17	QL17s9	QL49s9	SRS3596425	2016-07-16	52.51022 N 121.01133 W	335	572	59
QL17	QL17s10	QL49s10	SRS3596445	2016-07-16	52.51022 N 121.01133 W	329	536	61
QL15	QL15s1	QL52s1	SRS3596499	2016-07-18	52.50031 N 121.28803 W	441	876	50
QL15	QL15s2	QL52s2	SRS3596498	2016-07-18	52.50031 N 121.28803 W	407	760	54
QL15	QL15s3	QL52s3	SRS3596414	2016-07-18	52.50031 N 121.28803 W	400	830	48
QL15	QL15s4	QL52s4	SRS3596500	2016-07-18	52.50031 N 121.28803 W	396	812	49
QL15	QL15s5	QL52s5	SRS3596412	2016-07-18	52.50031 N 121.28803 W	371	739	50
QL15	QL15s6	QL52s6	SRS3596413	2016-07-18	52.50031 N 121.28803 W	373	745	50
QL15	QL15s7	QL52s7	SRS3596416	2016-07-18	52.50031 N	356	700	51

					121.28803 W			
QL15	QL15s8	QL52s8	SRS3596376	2016-07-18	52.50031 N 121.28803 W	341	673	51
QL15	QL15s9	QL52s9	SRS3596407	2016-07-18	52.50031 N 121.28803 W	342	638	54
QL15	QL15s10	QL52s10	SRS3596426	2016-07-18	52.50031 N 121.28803 W	338	652	52

Supplementary figure legend:

Figure S1 Concentration of various metals and metalloids, carbon and nitrogen, and the particle size composition of the sediment core samples. Sites were grouped to either Disturbed, Plume-influenced, or Natural (Undisturbed). Error bar represent standard deviation from the mean. Metal, metalloid, C, and N concentrations are in mg/Kg dry weight, D50 is the median particle size in μm, clay represents percent of particles between 0 μm – 2 μm in diameter, silt represents percent of particles between 2.01 μm and 62.5 μm in diameter, total sand represents particles >62.5 μm.

Figure S2 Optimal number of clusters based on metal data using k means clustering. Number of clusters to which samples clustered to was deduced based on the decay in decrease of within cluster sum of squares.

Figure S3 Dendrogram of samples based on UPGMA hierarchical clustering. Rectangles represent clusters based on k means clustering.

Figure S4 Relative abundance of class level taxonomic groups per sample, taxonomic groups with relative abundance lower than 5% of the total number of sequences\sample are represented by the others category. Others category contains 65 taxa.

Figure S5 Dendrogram of sample based on UPGMA hierarchical clustering rectangles represent clusters based on k means clustering

Figure S6 Positive co-occurrence network analysis, A) all positive (rho > 0.5) and statistically significant ($p<0.05$) co-occurrence patterns. B) Sub-network cluster of indicator OTUs. Degree centrality denotes number of edges connecting each node.

Figure S7 Optimal number of clusters based on OTU data based on k means clustering. Number of clusters to which samples clustered to was deduced based on the decay in decrease of within cluster sum of squares.

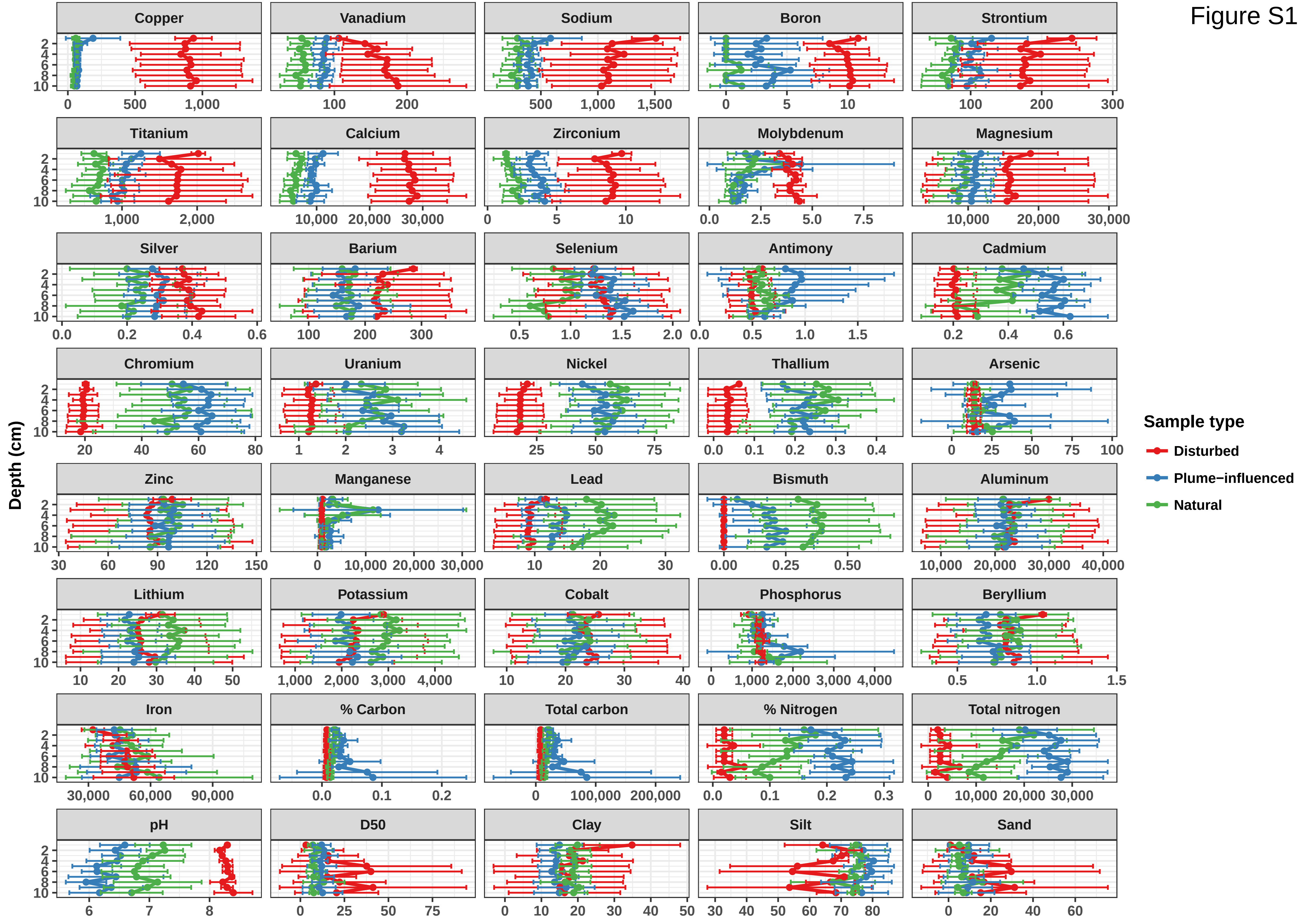
Figure S1
Copper
Vanadium
Sodium
Boron
Strontium
Titanium
Calcium
Zirconium
Molybdenum
Magnesium
Silver
Barium
Selenium
Antimony
Cadmium
Chromium
Uranium
Nickel
Thallium
Arsenic
Zinc
Manganese
Lead
Bismuth
Aluminum
Lithium
Potassium
Cobalt
Phosphorus
Beryllium
Iron
% Carbon
Total carbon
% Nitrogen
Total nitrogen
pH
D50
Clay
Silt
Sand
Depth (cm)
Sample type
Disturbed
Plume−influenced
Natural

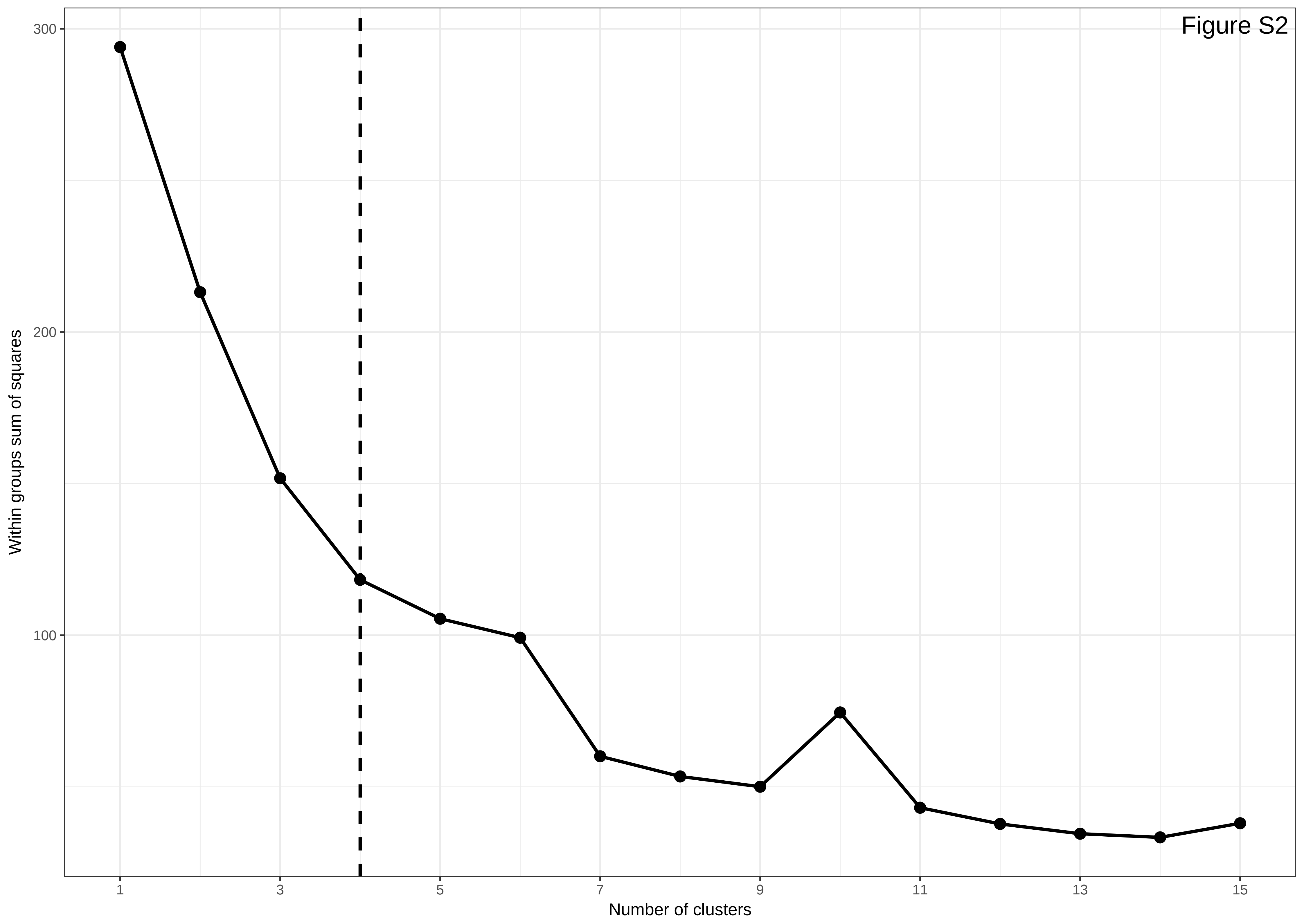
Figure S2
300
200
100
Within groups sum of squares
1
3
5
7
9
11
13
15
Number of clusters

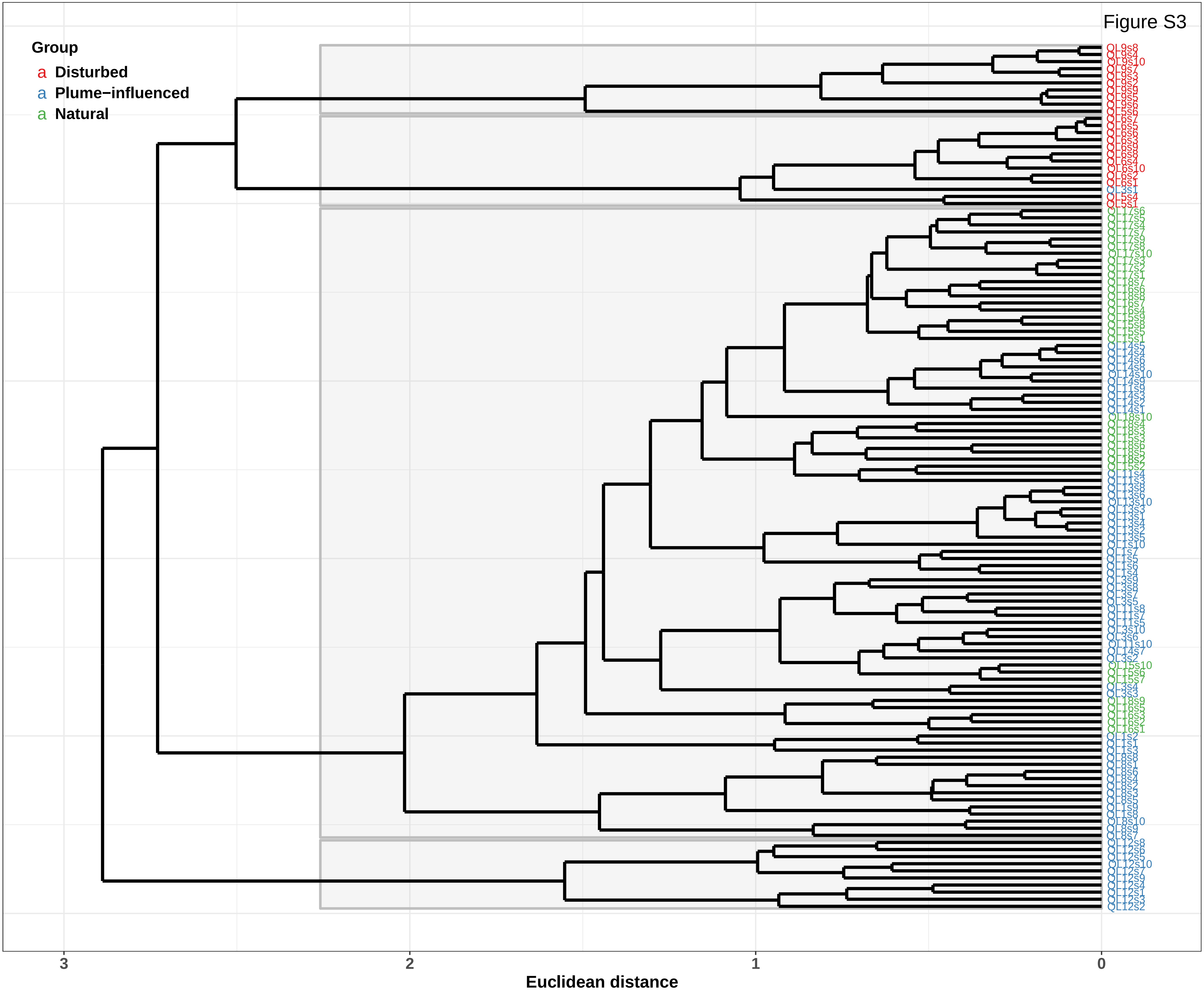
Group
a Disturbed
a Plume−influenced
a Natural
3
2
1
0
Euclidean distance
Figure S3

Figure S4

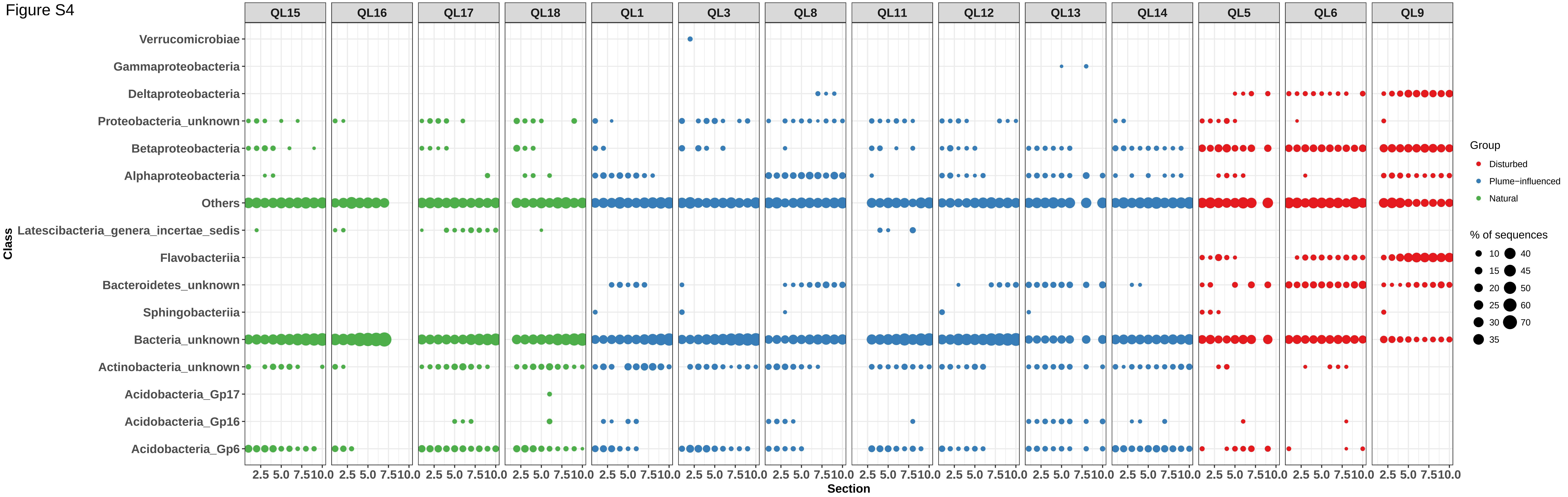

QL15
QL16
QL17
QL18
QL1
QL3
QL8
QL11
QL12
QL13
QL14
QL5
QL6
QL9
Verrucomicrobiae
Gammaproteobacteria
Deltaproteobacteria
Proteobacteria_unknown
Betaproteobacteria
Alphaproteobacteria
Others
Latescibacteria_genera_incertae_sedis
Flavobacteriia
Bacteroidetes_unknown
Sphingobacteriia
Bacteria_unknown
Actinobacteria_unknown
Acidobacteria_Gp17
Acidobacteria_Gp16
Acidobacteria_Gp6
Class
2.5 5.0 7.5 10.0
Section
Group
Disturbed
Plume−influenced
Natural
% of sequences
10
15
20
25
30
35
40
45
50
60
70

Figure S5

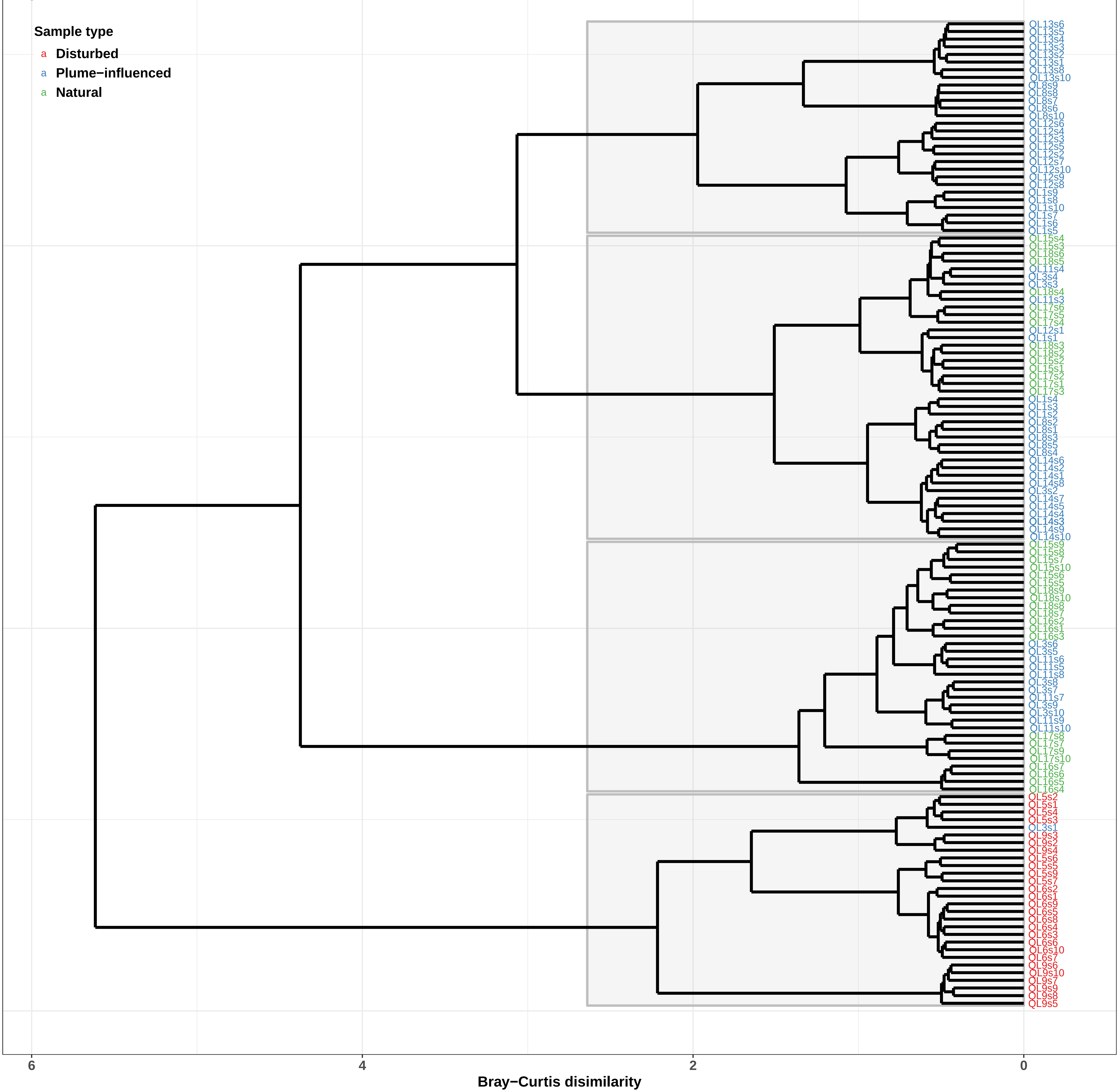
Sample type
a Disturbed
a Plume−influenced
a Natural
QL13s6
QL13s5
QL13s4
QL13s3
QL13s2
QL13s1
QL13s8
QL13s10
QL8s9
QL8s8
QL8s7
QL8s6
QL8s10
QL12s6
QL12s4
QL12s3
QL12s5
QL12s2
QL12s7
QL12s10
QL12s9
QL12s8
QL1s9
QL1s8
QL1s10
QL1s7
QL1s6
QL1s5
QL15s4
QL15s3
QL18s6
QL18s5
QL11s4
QL3s4
QL3s3
QL18s4
QL11s3
QL17s6
QL17s5
QL17s4
QL12s1
QL1s1
QL18s3
QL18s2
QL15s2
QL15s1
QL17s2
QL17s1
QL17s3
QL1s4
QL1s3
QL1s2
QL8s2
QL8s1
QL8s3
QL8s5
QL8s4
QL14s6
QL14s2
QL14s1
QL14s8
QL3s2
QL14s7
QL14s5
QL14s4
QL14s3
QL14s9
QL14s10
QL15s9
QL15s8
QL15s7
QL15s10
QL15s6
QL15s5
QL18s9
QL18s10
QL18s8
QL18s7
QL16s2
QL16s1
QL16s3
QL3s6
QL3s5
QL11s6
QL11s5
QL11s8
QL3s8
QL3s7
QL11s7
QL3s9
QL3s10
QL11s9
QL11s10
QL17s8
QL17s7
QL17s9
QL17s10
QL16s7
QL16s6
QL16s5
QL16s4
QL5s2
QL5s1
QL5s4
QL5s3
QL3s1
QL9s3
QL9s2
QL9s4
QL5s6
QL5s5
QL5s9
QL5s7
QL6s2
QL6s1
QL6s9
QL6s5
QL6s8
QL6s4
QL6s3
QL6s6
QL6s10
QL6s7
QL9s6
QL9s10
QL9s7
QL9s9
QL9s8
QL9s5
6
4
2
0
Bray−Curtis disimilarity

Figure S6a

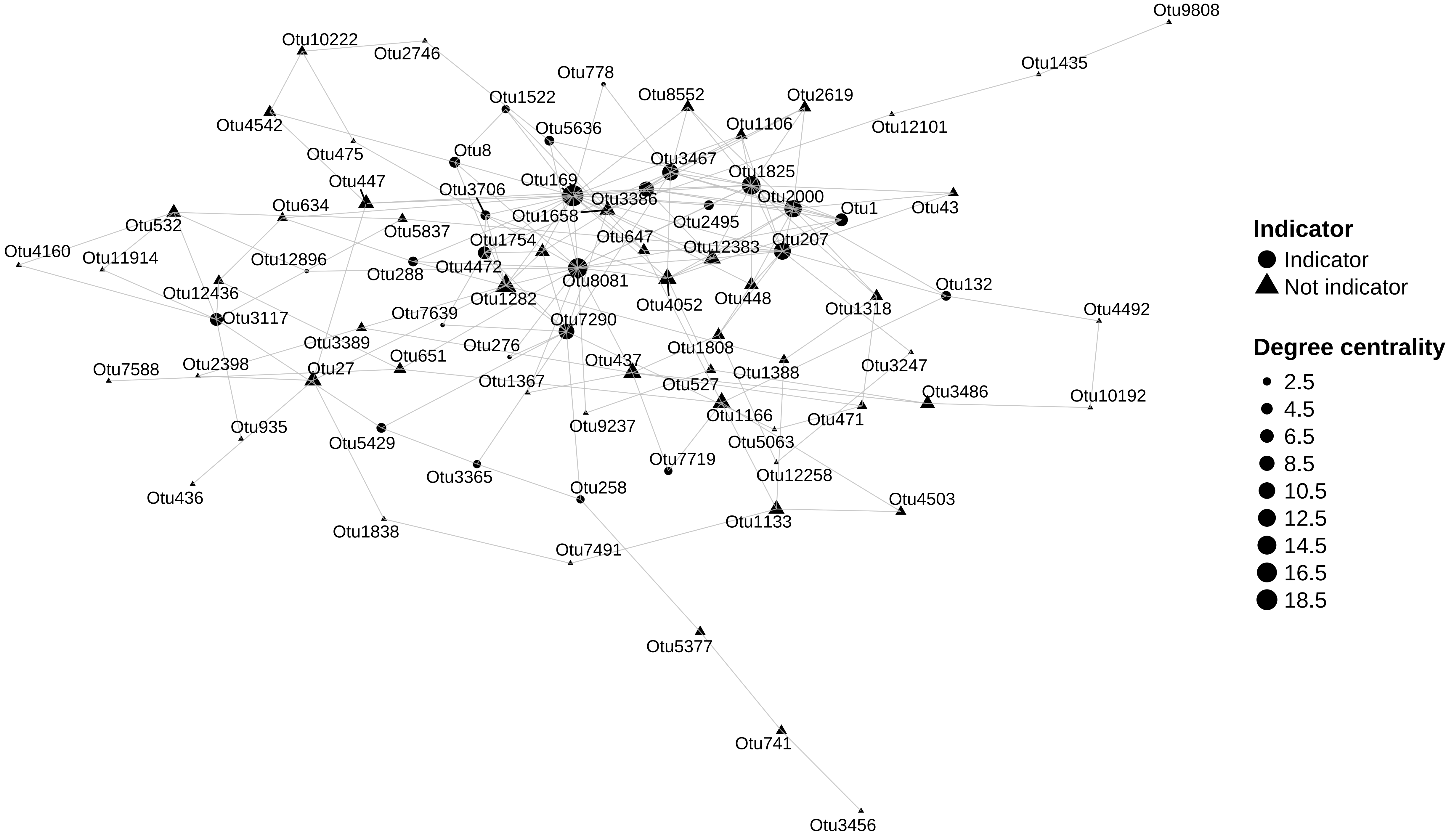
Otu9808
Otu10222
Otu2746
Otu1435
Otu778
Otu1522
Otu8552
Otu2619
Otu12101
Otu4542
Otu5636
Otu1106
Otu475
Otu8
Otu3467
Otu1825
Otu447
Otu169
Otu3706
Otu3386
Otu2000
Otu1
Otu43
Otu634
Otu1658
Otu532
Otu5837
Otu2495
Otu647
Otu1754
Otu12383
Otu207
Otu4160
Otu11914
Otu12896
Otu288
Otu4472
Otu8081
Otu132
Otu12436
Otu1282
Otu4052
Otu448
Otu1318
Otu4492
Otu7639
Otu3117
Otu7290
Otu3389
Otu276
Otu1808
Otu3247
Otu651
Otu437
Otu7588
Otu2398
Otu27
Otu1388
Otu1367
Otu527
Otu3486
Otu10192
Otu1166
Otu471
Otu935
Otu5429
Otu9237
Otu5063
Otu7719
Otu3365
Otu12258
Otu436
Otu258
Otu4503
Otu1133
Otu1838
Otu7491
Otu5377
Otu741
Otu3456
Indicator
Indicator
Not indicator
Degree centrality
2.5
4.5
6.5
8.5
10.5
12.5
14.5
16.5
18.5

Figure S6b

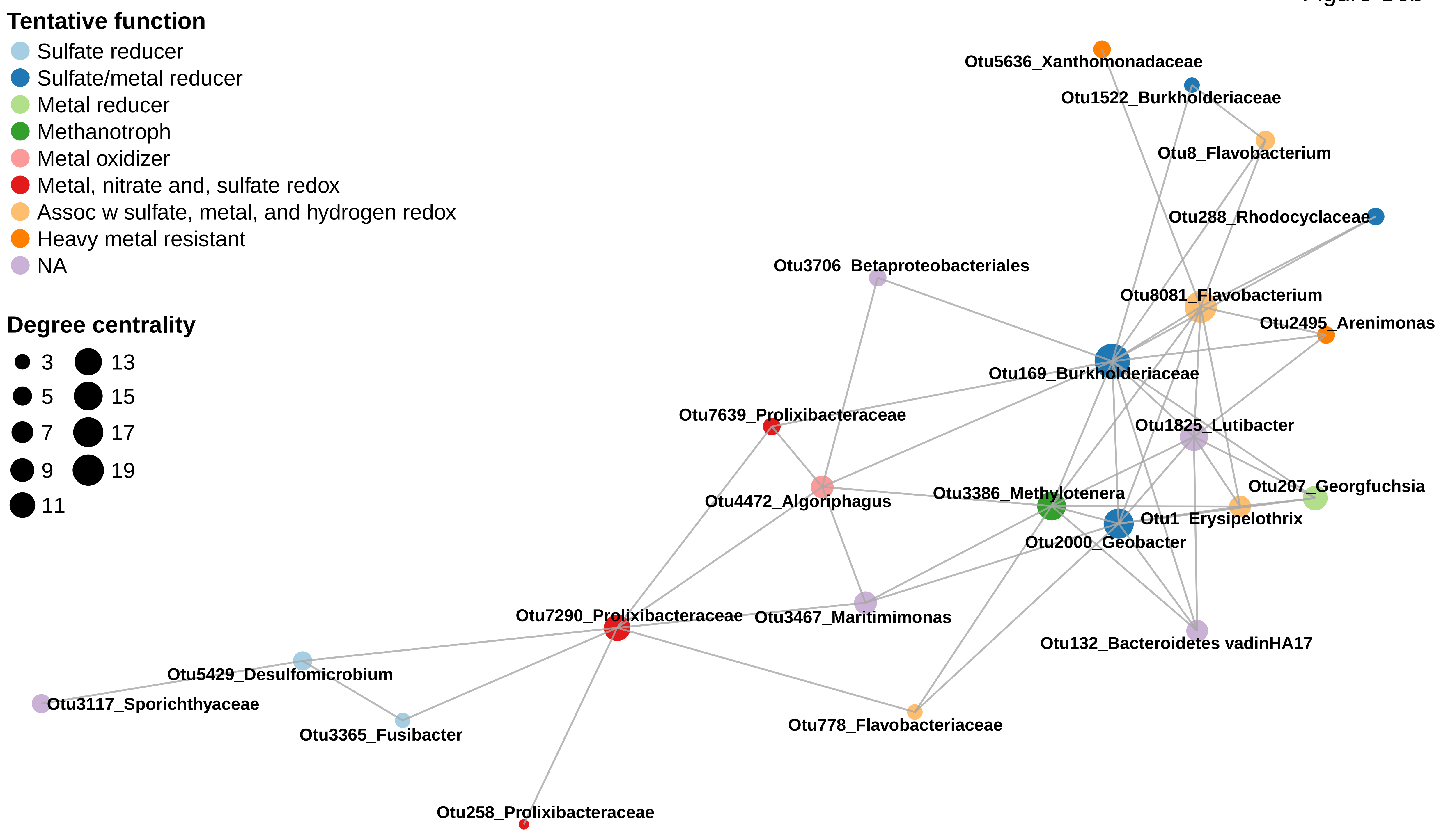
Tentative function
Sulfate reducer
Sulfate/metal reducer
Metal reducer
Methanotroph
Metal oxidizer
Metal, nitrate and, sulfate redox
Assoc w sulfate, metal, and hydrogen redox
Heavy metal resistant
NA
Degree centrality
3
5
7
9
11
13
15
17
19
Otu5636_Xanthomonadaceae
Otu1522_Burkholderiaceae
Otu8_Flavobacterium
Otu288_Rhodocyclaceae
Otu3706_Betaproteobacteriales
Otu8081_Flavobacterium
Otu2495_Arenimonas
Otu169_Burkholderiaceae
Otu7639_Prolixibacteraceae
Otu1825_Lutibacter
Otu4472_Algoriphagus
Otu3386_Methylotenera
Otu207_Georgfuchsia
Otu1_Erysipelothrix
Otu2000_Geobacter
Otu7290_Prolixibacteraceae
Otu3467_Maritimimonas
Otu132_Bacteroidetes vadinHA17
Otu5429_Desulfomicrobium
Otu3117_Sporichthyaceae
Otu3365_Fusibacter
Otu778_Flavobacteriaceae
Otu258_Prolixibacteraceae

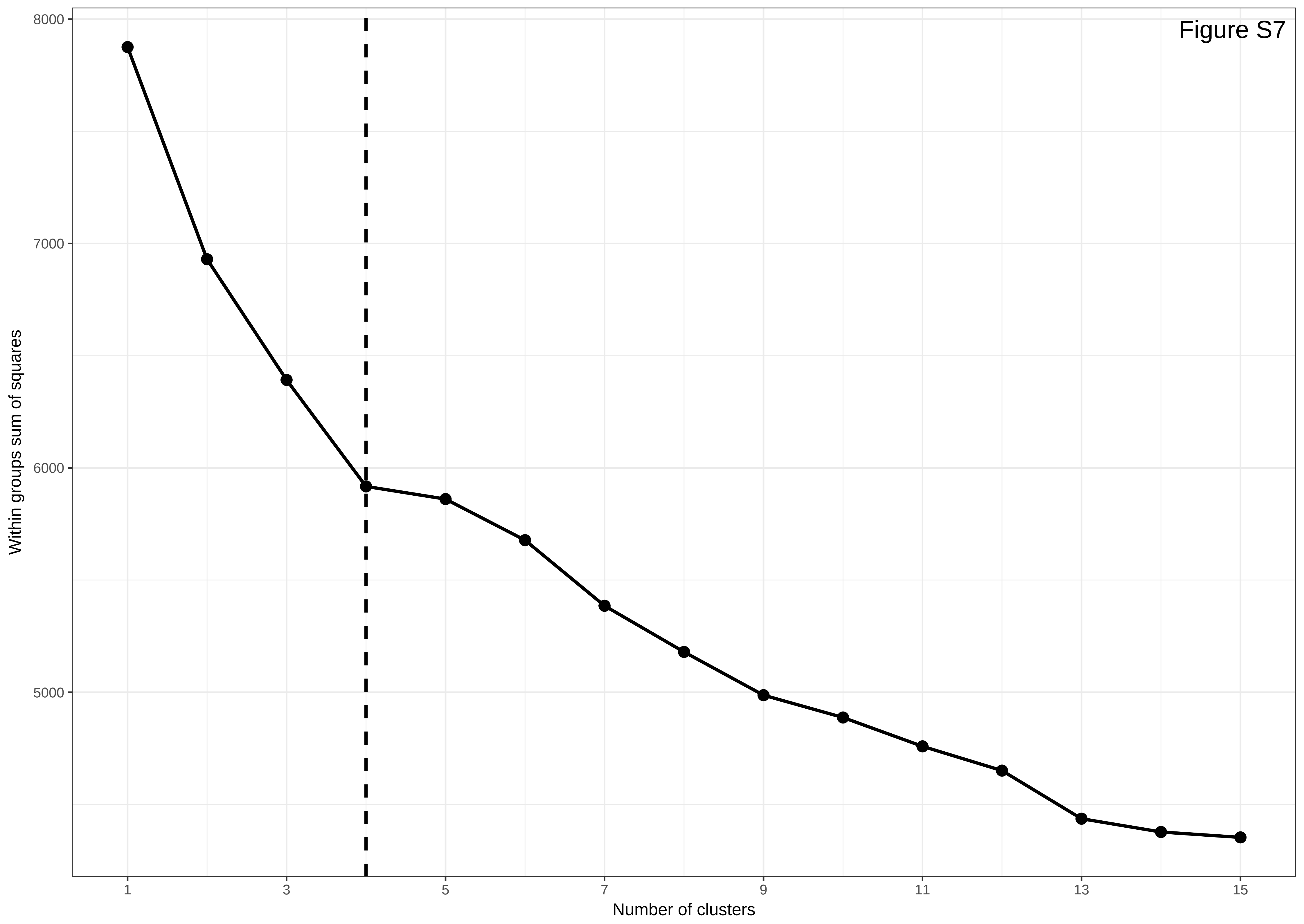
Figure S7
Within groups sum of squares
8000
7000
6000
5000
1
3
5
7
9
11
13
15
Number of clusters
